# Continuous glucose monitoring metrics and pregnancy outcomes in women with gestational diabetes: a secondary analysis of the DiGest trial

**DOI:** 10.2337/dc25-0452

**Published:** 2025-08-19

**Authors:** Laura C Kusinski, Nooria Atta, Danielle L. Jones, Suzanne Smith, Louise Cooper, Linda M. Oude Griep, Kirsten Rennie, Emanuella De Lucia Rolfe, Helen R Murphy, Eleanor M Scott, Stephen J. Sharp, Roy Taylor, Claire L Meek

**Affiliations:** 1 Leicester Diabetes Centre and Leicester NIHR Biomedical Research Centre, University of Leicester, University Hospitals Leicester, Gwendoline Road, Leicester LE5 4PW, UK; 2 Institute of Metabolic Science- Medical Research Laboratories, Cambridge Biomedical Campus, Hills Road, University of Cambridge, CB2 0QQ UK; 3 MRC Epidemiology Unit, Cambridge Biomedical Campus, Hills Road, University of Cambridge, CB2 OQQ UK; 4 University of East Anglia, Bob Champion Building, Research Park, Rosalind Franklin Road, Norwich, NR4 7UQ, UK; 5 Leeds Institute of Cardiovascular and Metabolic Medicine, School of Medicine, University of Leeds, Clarendon Way, Leeds, LS2 9JT, UK; 6 Newcastle University, Health Innovation Neighbourhood, Newcastle, NE4 7UQ, UK

**Keywords:** Continuous glucose monitoring, gestational diabetes mellitus, time in range, time above range, time below range, pregnancy outcomes

## Abstract

**Objective:**

Continuous glucose monitoring (CGM) is increasingly used in gestational diabetes mellitus (GDM) but optimal metrics, ranges and targets in this population are undefined. We assessed associations between CGM metrics and pregnancy outcomes in gestational diabetes.

**Research Design and Methods:**

During the DiGest study, 425 women with GDM (diagnosed at median (IQR) 25.1 (18.3- 27.7) weeks) and BMI ≥25kg/m^2^ received a dietary intervention, with masked Dexcom G6 CGM at 29 (n=361), 32 (n=215) and 36 weeks (n=227) gestation. For this secondary analysis, we used logistic regression, receiver-operator-curves and Youden index to assess associations and predictive ability of CGM metrics including pregnancy-specific time-in-range (TIRp; 63-140mg/dL; 3.5-7.8mmol/L) and pregnancy outcomes.

**Results:**

CGM metrics at 29 weeks were significantly associated with LGA and SGA. Participants achieving mean glucose <110mg/dL (6.1mmol/L), TIRp ≥90% or pregnancy-specific time-above-range (TARp) <10% at 29 weeks had a significantly lower risk of LGA (OR 0.41 (95%CI 0.22-0.77); OR 0.38 (0.20-0.70); OR 0.39 (0.20-0.73) and SGA (OR 0.26 (0.08-0.79); OR 0.30 (0.10-0.91); OR 0.19 (0.06-0.62)). TARp<10% and mean nocturnal glucose <110mg/dL were associated with reduced odds of preterm birth (OR 0.40 (0.17-0.94); OR 0.42 (0.19-0.97)). A stricter range (63-120mg/dL; 3.5-6.7mmol/L) had similar performance overall, but had no single statistically-robust TIR/TAR target across all outcomes.

**Conclusions:**

In women with GDM, CGM mean glucose <110mg/dL (6.1mmol/L), ≥90% TIRp or <10% TARp using a range of 63-140mg/dL (3.5-7.8mmol/L) at 29 weeks of gestation was associated with a low risk of suboptimal offspring outcomes.

## Introduction

Gestational diabetes mellitus (GDM) affects around 14% of pregnancies worldwide (1) and is associated with suboptimal perinatal outcomes and increased risk of future cardiometabolic disease (2–4). With rising GDM prevalence internationally, there is an urgent need to improve short-term outcomes and reduce the lifelong burden of chronic disease.

Optimizing glycemic control is the key management strategy to reduce the perinatal complications of GDM (3). Real-time continuous glucose monitoring (CGM) improves pregnancy outcomes in women with type 1 diabetes in pregnancy (5,6). who are recommended to attain ≥70% pregnancy-specific time in range (TIRp) using a range of 63-140mg/dL (3.5-7.8mmol/L) (7). However, it is unclear if a similar glucose target range and risk thresholds are appropriate in GDM, a condition usually characterised by milder degrees of hyperglycemia (3,6). Indeed, recent evidence suggests that more stringent glycemic targets (6,8), especially for overnight glycemic control (9), might be more appropriate for clinical management of GDM.

In this analysis we assessed associations between CGM metrics and pregnancy outcomes in women with GDM in order to identify candidate CGM metrics for clinical use.

## Research Design and Methods

In this pre-specified secondary analysis, we used data collected during the Dietary Intervention in Gestational diabetes (DiGest) trial, a randomized, controlled, double-blind whole-diet intervention trial conducted in eight hospital centers in England, funded by Diabetes UK. Dexcom Inc supplied CGM equipment. Funder did not influence the trial design, conduct or reporting. The DiGest trial was approved by the National Research Ethics Committee, UK (reference 18/WM/0191) and the NHS Health Research Authority (IRAS 242924; ISRCTN 65152174). The trial protocol and results have been published previously (10,11).

Due to the Covid-19 pandemic, adjustments were made to the original DiGest protocol. Pregnant women aged ≥18 years old with a diagnosis of GDM (NICE guidelines or Royal College of Obstetricians and Gynaecologists interim Covid-19 criteria during 2020-2022; random glucose 162-200mg/dL (9-11mmol/L) or HbA1c 41-47mmol/mol at booking; fasting glucose ≥100mg/dL (≥5.6mmol/L) or Hba1c ≥39mmol/mol (10,12), before 30+6 weeks’ gestation, and a BMI ≥25 kg/m^2^, were eligible to participate in the trial. Exclusion criteria included multiple pregnancy, complications such as threatened preterm labour, severe anemia, intrauterine growth restriction at baseline, or evidence of severe congenital abnormality on ultrasound. Women were also ineligible if they had a hemoglobin A1c (HbA1c) ≥6.5% (48 mmol/mol) at GDM diagnosis, a history of diabetes outside pregnancy, or were using medications (e.g. high dose oral steroids) that could interfere with OGTT results.

Trial participants were randomly allocated to receive either a reduced-energy diet (1200kcal/day) or a standard-energy diet (2000kcal/day) until delivery, supplied by dietboxes containing 40% carbohydrate, 35% fat, 25% protein with low-glycemic index foods only. Participants and clinical teams were blinded to the intervention allocation. GDM management at all centers followed the NICE guidelines which included self-monitoring blood glucose four times daily with thresholds of <95mg/dL (<5.3 mmol/L) fasting, <140mg/dL (<7.8 mmol/L) 1-hr post-prandially, dietary modification, followed by medications (metformin/ insulin) for women with persistent hyperglycemia (13,14). Since the intervention did not significantly change primary outcomes or antenatal glycemia, we have used the data as a cohort study for the purposes of this analysis.

### Trial visits and CGM data collection

A masked CGM monitor (Dexcom G6) was positioned on the participant’s arm (usually non-dominant upper arm) at ~29, 32 and 36 weeks and worn consecutively for 7-10 days. CGM devices which were dislodged early were re-sited, where possible.

CGM data at 29 weeks reflect women’s habitual diet following a diagnosis of GDM after standard nutritional advice from healthcare teams; the dietary intervention started one week later.

Participants’ clinical data and HbA1c values were extracted from their medical records where available (note reduced blood testing during Covid-19 in all centers).

### Study outcomes

LGA was the primary outcome of this analysis. We included pre-specified pregnancy outcomes from the core outcome set for GDM (15). These include preeclampsia (blood pressure ≥140/90 mmHg and proteinuria or clinical or laboratory evidence of end-organ damage) (16), preterm birth (delivery <37 weeks’ gestation), large-for-gestational-age (LGA; >90% INTERGROWTH centiles adjusted for gestational age, baby sex and ethnicity), small-for-gestational-age (SGA; <10% INTERGROWTH centiles), admission to the neonatal intensive care unit (NICU) and neonatal hypoglycemia (capillary glucose <2.6mmol/L on one or more occasions, within the first 48-hours, starting ≥30 minutes post-birth)(10).

### CGM data processing and ranges

Raw CGM data were downloaded from Dexcom Clarity and metrics calculated in line with the international consensus statement (7), including pregnancy-specific time-in-range (TIRp; 63-140mg/dL (3.5-7.8mmol/L)), time-above-range (TARp; >140mg/dL (7.8mmol/L)), time-below-range (TBRp; <63-mg/dL (3.5mmol/L)), mean CGM glucose, standard deviation (SD) and coefficient of variation (CV) of CGM glucose.

Recent work has suggested a stricter glucose range “stringent TIRp” of 63-120mg/dL (3.5-6.7mmol/L) or use of nocturnal metrics (00:00h to 06:00h) (9,18) for women with GDM (8,17) which were also calculated for comparison.

Women with at least 24-hours of CGM data were eligible for inclusion in this analysis. We included CGM data recorded from 00:00 hours on the day following CGM application, excluding any data recorded earlier. In a sensitivity analysis, we included data from women with at least 96-hours of CGM data and presented the results in the supplementary material.

### Statistical Analysis

The data are presented as mean (SD) or proportions as appropriate. Multivariable logistic regression was used to examine the association with the CGM metrics and the outcomes.

Maternal outcomes were adjusted for the trial arm, maternal age, BMI at enrolment, ethnicity, parity (primiparous yes/no) and education (degree yes/no). In addition, gestational age at delivery and infant sex were also included in the adjustments for neonatal outcomes to evaluate the independent role of CGM metrics on those outcomes. The results are reported as adjusted odds ratios (OR) with 95% confidence intervals (CIs) and p<0.05. Where appropriate, the CGM metrics are standardized and the OR (95% CI) per standard deviation change in the metrics are presented.

Receiver operating characteristics (ROC) analysis was used to assess the predictive performance of mean glucose and TIRp, TARp and stringent metrics comparatively. For each CGM metric, the area under the curve (AUC) was calculated (19). The Youden index (sensitivity + specificity -1) was used to identify the optimal cut-off points for the CGM metrics (20). Based on the thresholds identified, we compared pregnancy outcomes among those who achieved the glycemic targets and those who did not. The results are presented as CGM targets and CGM markers of increased risk for pregnancy complications. Participants with missing CGM data were removed from the analysis.

Statistical significance was defined as two-sided p≤0.05. The analyses were conducted in SPSS Statistics v.29 (IBM Corp. Released 2022. IBM SPSS Statistics for Windows, Version 29.0; IBM Corp) and STATA (version 18.0; StataCorp).

## Results

### Characteristics of the participants

The DiGest Trial recruited 425 women between November 2019 to July 2023. In this study, women with at least 24-hours of CGM data at any time point (n=379) are included (Table 1 and Supplementary Figure 1). Baseline characteristics of women with and without 96-hours of CGM data are presented in Supplementary Tables 1. CGM metrics at baseline, 32 and 36 weeks are provided in Table 1 and Supplementary Tables 2, 3 and 4. Women with diagnosis before 20^th^ weeks of gestation (28.7%; supplementary figure 2) had higher rates of medications at recruitment (Supplementary Table 5).

Among women included in this cohort, 6/351 (1.7%) had preeclampsia, 31/352 (8.8) babies were born before 37 weeks of gestation, 63/350 (18.0%) newborns were LGA, and 16/350 (4.6%) babies were SGA. 31/350 babies (8.9%) were admitted to NICU and 17/350 (4.9%) had neonatal hypoglycemia (Table 1).

Due to the small numbers of women with preeclampsia, reporting has focussed on other outcomes (see Supplementary Tables 6-7).

### Pregnancy outcomes and CGM metrics at 29 weeks

Mean CGM glucose, TIRp and TARp at 29 weeks of gestation were associated with fetal growth outcomes (Table 2). Standardized mean glucose was associated with LGA (OR 1.57; 95%CI 1.16-2.13) and SGA (OR 1.89; 95%CI 1.10-3.24; remaining significant after additional adjustment for metformin). One standard deviation (SD) increase in TIRp or a one SD increase in stringent TIRp was associated with reduced odds of LGA (OR 0.54; 95%CI 0.40-0.73 and 0.66; 95%CI 0.50-0.87 respectively), while TARp and stringent TARp increased the odds of LGA (OR 1.80; 95%CI 1.34-2.41 and 1.49: 95%CI 1.13-1.97 for a one SD increase respectively). Similar findings were observed for nocturnal metrics (Table 2).

There was no association between any of the CGM metrics at 29 weeks of gestation and preterm delivery, NICU admission and neonatal hypoglycemia. TBRp was not significantly associated with pregnancy outcomes at 29 weeks (Table 2). Caesarean delivery was not associated with any CGM metrics at any timepoint (data not shown).

Glucose variability was also significantly associated with LGA risk. Both glucose SD (OR 7.17; 95%CI 2.53-20.32) and coefficient of variation (CV) (OR 1.10; 95%CI 1.02-1.19) at 29 weeks were significantly associated LGA (Table 2), which remained significant after adjusting for mean glucose (OR 5.12; 95%CI 1.43-18.26) (Table 2).

A sensitivity analysis in women with pharmacologic treatment at recruitment showed comparable results, with the effect size for a majority of the CGM metrics being increased in these participants (Supplementary Table 8).

### CGM metrics and pregnancy outcomes at 32-36 weeks

Associations between CGM metrics and pregnancy outcomes at 32 and 36 weeks of gestation were similar (Supplementary Tables 9-10). At 32 weeks, mean glucose and standard TARp were associated with LGA (OR 1.57 95%CI 1.09-2.26 and OR 1.42 95%CI 1.01-2.01 respectively). TBRp at 32 weeks was inversely associated with LGA (OR 0.11 95%CI (0.02-0.64). At 36 weeks, mean glucose,TIRp and TARp using both standard and stringent ranges were associated with LGA, but associations were stronger for metrics using the standard range (Supplementary Table 10). NICU admission and neonatal hypoglycemia were associated with TBRp at 36 weeks of gestation (Supplementary Table 10).

### ROC analysis for association of CGM metrics at 29 weeks with pregnancy outcomes

**Figure 1:**
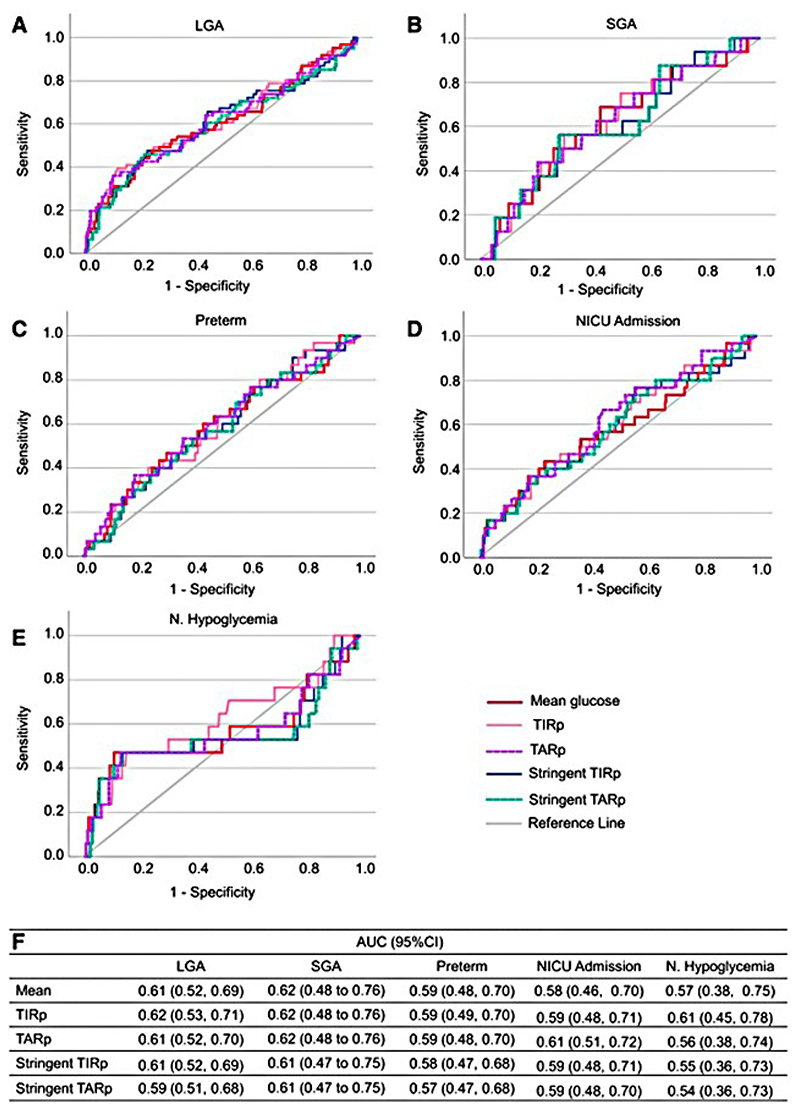
ROC analysis of CGM mean glucose, TIR and TAR at 29 weeks of gestation and pregnancy outcomes. Pregnancy outcomes include LGA (A), SGA (B), preterm birth (C), NICU admission (D) and neonatal hypoglycemia (E). Area under the curve analysis for each of the parameters and the pregnancy outcomes are shown in F.

The ROC analysis results for associations of CGM metrics with pregnancy outcomes are shown in Table 3, Supplementary Table 11, Figure 1 and Supplementary Figure 1.

#### LGA and SGA

Mean glucose, TIRp stringent TIRp, TARp and stringent TARp were significant predictors of LGA in women with GDM (Table 3). The optimal threshold for prediction of LGA was TIRp ≥93.5% and TARp <3.5% and stringent TIRp ≥80.1% and stringent TARp <16.2% (Table 3 and Figure 1). The ROC analysis results for nocturnal CGM metrics were consistent with the 24-hours results (Table 3 and Supplementary Figure 3).

#### NICU admission

Most CGM metrics did not significantly predict NICU admission (Table 3 and Figure 1). However, TARp was the only CGM metric associated with NICU admission (AUC 0.61; 95% CI 0.51-0.72). The optimal threshold for prediction of NICU admission was TARp <3.6% (Table 3 and Figure 1).

The ROC analysis highlighted that, on average, the optimal threshold of TIRp ≥90% and stringent TIRp ≥70% are associated with reducing the risk of suboptimal pregnancy outcomes, such as LGA. An average threshold of TARp ≥10% and stringent TARp ≥30% were related to predicting LGA and NICU admission (Table 3).

#### Preterm delivery and neonatal hypoglycemia

No CGM metrics were significant predictors of preterm delivery or neonatal hypoglycemia on ROC analysis (Table 3 and Figure 1).

#### Preeclampsia

Preeclampsia showed significant associations with CGM metrics but had very few events (Supplementary Table 11;Supplementary Figure 4).

### Setting recommendations for CGM targets in GDM

In this analysis, mean glucose thresholds of 110-113mg/dL (6.1-6.3mmol/L) were suggested as the optimal targets for risk prediction of LGA or preeclampsia (Table 3 and Supplementary Table 11). Using a clinical management target of mean glucose <110mg/dL (6.1mmol/L) would identify around 25.8% of the population who needed more intensive support, since 74.2% of our study participants had a mean glucose lower than 110mg/dL (6.1mmol/L) at enrolment (Table 1).

In this study, TIRp ≥90.0-93.5% and TARp <3.5-10.0% were suggested as the optimal thresholds for risk prediction (Table 3 and Supplementary Table 11). Using a threshold of TIRp ≥93.5% would identify around 43% of the population who needed more intensive support, since 57% of our study participants met this threshold at enrolment. Using a target of TIRp ≥90% would identify around 27.1% of the population who needed more intensive support, since 72.9% of our study participants met this threshold at enrolment (Table 1). Moreover, 68% of participants had achieved both TIRp ≥90% and mean glucose <110mg/dL (6.1mmol/L) (Supplementary Figure 5). We compared the performance of a TIRp threshold of 90 or 94% (Table 2) and a 90% threshold was able to predict more outcomes significantly. We compared the performance of a TARp threshold of 4 or 10% (Table 2) and a 10% threshold was able to predict more outcomes significantly.

Using the stringent range, our ROC analysis suggested the TIRp ≥59.4-80.1% and TARp <16.2-31.0% as the optimal thresholds for risk prediction (Table 3;Supplementary Table 11). Using a threshold of stringent TIRp ≥70% or TARp <30% would each identify around 23.8% of the population who needed more intensive support, since 76.2% and 76.5% of our study participants met these thresholds (TIRp ≥70% and TARp <30% respectively) at enrolment (Table 1).

### Performance of suggested CGM thresholds in GDM

Participants who achieved the mean glucose target of <110mg/dL (6.1mmol/L) at 29 weeks had a significant reduction in the rates of LGA (OR 0.41; 95% CI 0.22-0.77) and SGA (OR 0.26; 95% CI 0.08-0.79; Table 2). Meanwhile women who achieved TIRp ≥90% or TARp <10% had lower risk of LGA (OR 0.38; 95% CI 0.20-0.70 and OR 0.39; 95% CI 0.20-0.73 respectively) and SGA (OR 0.30; 95% CI 0.10-0.91 and OR 0.19; 95% CI 0.06-0.62 respectively; Table 2). Similar associations were found for CGM metrics using the stringent range or at night (Table 2). Women who achieved both TIRp ≥90% and mean <110mg/dL (6.1mmol/L) had reduced risk of LGA and SGA (Supplementary Table 12). Achieving a TARp <10% and nocturnal mean glucose of <110mg/dL (6.1mmol/L) was associated with lower risk of preterm birth (OR 0.40; 95% CI 0.17-0.94 and OR 0.42; 95% CI 0.19-0.97, respectively). Results for preeclampsia are provided in Supplementary Table 6.

Results were comparable in a cohort of women with >96 hours of CGM data (Supplementary Tables 13-15;Supplementary Figures 6-7) and in women receiving pharmacological treatment at recruitment (Supplementary Figure 8).

## Discussion

### Statement of principal findings

In women with GDM, CGM indicators of hyperglycemia, including TIRp, TARp and mean glucose at 29 weeks of gestation are predictive of pregnancy growth outcomes, particularly LGA and SGA. Our data confirm that CGM is a valuable risk stratification tool at GDM diagnosis. Based on our data, we put forth provisional targets of mean CGM glucose <110mg/dL, ≥90% TIR and <10% TARp using the standard pregnancy range (63-140mg/dL; 3.5-7.8mmol/L) for the management of GDM.

### Strengths and weaknesses

The DiGest trial was a randomised controlled double-blind study of a whole-diet reduced energy intervention, with rich CGM data at 29-, 32, and 36 week’s gestation. The study captured a wide range of maternal demographics and detailed maternal and neonatal data making this a strong dataset to analyse. Data recorded at 29 weeks’ captures habitual diet, whereas the participants were supplied with their study diet thereafter. Providing the food to participants is a strength, as controlling the confounder of food, allows us to assess associations between CGM metrics and outcomes. Indeed, provision of homogenous low glycemic food supply at 32 and 36 weeks may have been a limitation, as may have improved glucose concentrations at those timepoints, leading to improved outcomes and weaker associations between CGM metrics and outcomes. However, macronutrient composition at 29 weeks was similar to that provided at 32 and 36 weeks (Jones et al., manuscript in preparation).

Participants were recruited in eight health centers in England, who followed the same national care pathway, but minor variations in local practices occurred during the Covid-19 pandemic. Different sites had different approaches for identification of early GDM. Medication at the time of enrolment may reduce the associations between glycaemia and outcomes. All participants had a BMI ≥25 kg/m^2^ which might reduce the applicability of our findings to lean women. Our trial population was more diverse than the UK population, but still included relatively low numbers of women from Asian and Black ethnic groups.

Trial participants had low rates of preeclampsia, preterm birth, SGA and neonatal hypoglycemia compared to the general population of women with GDM (21) (22)which may have led to instability in the statistical models, warranting caution in interpreting the data for the less common outcomes. We acknowledge that outcomes such as LGA are multifactorial, and predictive models including maternal BMI, gestational weight gain, ethnicity, parity and socioeconomic status may provide higher AUCs. However, the aim of our study was to assess CGM metrics as predictors of pregnancy outcomes in GDM. Future work to build more complete multivariate models is under consideration.

Timing of GDM diagnosis has recently been found to be important in determining outcomes. However, in this cohort, women with early (diagnosed <20 weeks) vs standard diagnosis of GDM had comparable CGM metrics at 29, 32 and 36 weeks and comparable pregnancy outcomes, so we did not adjust for timing of diagnosis in our analysis (manuscript in preparation). The relevance of early-pregnancy CGM metrics in diagnosing GDM is being examined more fully elsewhere and was the aim of this study (23,24).

Another limitation is the optimal CGM metric thresholds were derived and tested in the same population cohort. These findings need to be validated in future cohorts to determine if these target thresholds are useful or whether they need to be further refined. CGM was tested after diagnosis when women might have already changed their diet, resulting in weakening of associations.

### Which CGM metrics are most important for risk stratification in GDM?

In women with GDM, TIRp, TARp and average CGM glucose were all associated with LGA and SGA. The results of logistic regression and ROC analysis showing associations between CGM metrics and LGA are consistent in size and direction with our previous work in women with type 1 diabetes in pregnancy (25) and other work in women with pregestational diabetes (26) or women at risk of GDM (27). However, we did not find frequent associations between CGM metrics and other important perinatal outcomes of GDM, including preterm delivery, neonatal hypoglycemia and NICU admission, perhaps due to small number of these complications in trial participants, or different data collection timepoints.

Our data show an association between elevated maternal mean CGM glucose and SGA. While SGA reflecting insufficient placentation and poor placental function has been observed in type 1 diabetes (28), it has not been commonly described in GDM. However, Li and colleagues identified that raised 0-hour and 2-hour results from a 75g OGTT and raised 2-hour postprandial glucose and HbA1c in the second trimester were risk factors for SGA in 1910 women with GDM in China (29). Durnwald and colleagues assessed associations between CGM metrics and SGA in a mixed cohort of women with and without GDM and identified that CGM metrics associated with lower mean glucose and hypoglycemia were associated with SGA (27). This might suggest that the causes of SGA in a predominantly normoglycemic population are quite different to those seen in a hyperglycaemic population. It is unclear why SGA rates were so low in this predominantly normoglycemic population (27).

### Which glucose targets are the most appropriate for use in GDM?

Our findings highlight the value of CGM metrics at 29 weeks for predicting the risk of both LGA and SGA. Randomised controlled studies are needed to show if CGM as self-management tool improves outcomes, supported by clear glycaemic targets. Our data offer evidence to support a provisional target range for glycemia in GDM, which can be assessed and refined in future trials.

The optimal glucose range for use in GDM management is unclear. Recent studies (8,17,23,24) have investigated which CGM metrics could be used for GDM diagnosis. These authors found that a stringent TIRp (63-120mg/dL (3.5-6.7mmol/L) was likely to be superior for prediction of GDM diagnosis compared to the TIRp of 63-140mg/dL (3.5-7.8mmol/L) (8).

Our data suggest that the standard pregnancy range was slightly superior than the stringent range for risk prediction in GDM. Using the standard range, TIR or TAR were significantly associated on regression or ROC analysis with three outcomes (LGA, SGA and NICU admission). The corresponding ROC thresholds for these outcomes were 90.7-95.6% for TIR and 3.5-10.6% for TAR with 90% TIR and 10% TAR for preeclampsia (albeit with small numbers). We chose a target of >90% TIR and <10% TAR which would have adequate performance for all outcomes, and which identified around 25% of women as a higher risk group, needing treatment escalation to reach these targets. Conversely, TIR and TAR using the stringent pregnancy range showed significant associations on regression or ROC analysis for two outcomes (LGA and SGA). The corresponding ROC thresholds for these outcomes were 74.2-80.1% for TIR and 16.2-23.9% for TAR. In addition, preeclampsia was significantly associated with an optimum threshold of 60% TIR and 31% TAR (small numbers). These thresholds for the stringent range were less consistent, making it more difficult to choose a single robust threshold point. We therefore concluded that attaining targets of TIRp ≥90% and TARp <10% using the standard pregnancy range are useful for risk stratification and may be useful for management, with further potential benefits associated with TIRp >94% and TARp <4% are achieved. Using the stringent range, thresholds of TIRp ≥70% and TARp <30% identified a similar number of patients, but need further study to refine the optimal thresholds. A larger dataset with more cases of preeclampsia, neonatal hypoglycemia and NICU admission would be needed to make a robust comparison of multiple different glucose ranges.

### Which risk thresholds or management targets should be used in GDM?

An RCT is required to establish clear management targets in women with GDM, however, our data suggest that attaining pregnancy targets of mean glucose <110mg/dL (6.1mmol/L), TIRp ≥90% or TARp <10% are likely to be useful for management, with further benefits evident (reduced LGA and NICU admission) if TIRp >94% and TARp <4% are achieved. Our suggested targets might provide a useful resource to support RCT design.

Targets are important for supporting self-management of GDM, addressing maternal anxiety and helping clinicians prioritise patients who need additional support to improve glycemia. There is a balance between choosing targets which offer “perfect” glucose control but may be unattainable to the majority of patients and choosing targets which are pragmatic and helpful on a population/ clinical service level. Targets should allow the highest risk patients to be easily identified and prioritised for interventions. In our cohort, around 75% of patients were able to achieve these targets of mean glucose <110mg/dL (6.1mmol/L), TIRp ≥90% or TARp <10% at enrolment, suggesting that these are attainable targets for the majority of women with GDM. The 25% of patients who did not achieve targets at 29 weeks could be prioritised for more regular appointments, further dietitian input, medication and enhanced screening for complications such as LGA or SGA.

### Future directions and implications for clinical care

A growing body of evidence suggests that CGM use in women with GDM is associated with better glycemic control and improved outcomes (30,31), improved quality of life and improved adherence to dietary strategies or pharmacotherapies (30–33). The DiGest trial did not assess the value of unmasked CGM as a self-management tool in GDM. However, our study suggests that attaining >90% pregnancy TIR (63-140 mg/dL; 3.5-7.8 mmol/L) was associated with improved pregnancy outcomes in women with GDM. Our recent qualitative study in women at risk of GDM found that CGM use was highly acceptable in pregnancy (34). Further validation studies may clarify if derived thresholds are applicable across different CGM devices (6).

## Conclusion

In women with GDM, attaining CGM mean glucose <110mg/dL (6.1mmol/L), ≥90% TIRp or <10% TARp using a range of 63-140mg/dL (3.5-7.8mmol/L) at 29 weeks of gestation was associated with a low risk of suboptimal offspring outcomes. An RCT is required to identify if CGM is a useful tool in self-management of GDM and to definitively assess individual glucose ranges and targets.

## Supplementary Material

Supplementary Tables and Figures

## Figures and Tables

**Table 1:** Baseline characteristics of participants at enrolment. Results are presented as mean (SD) or n (%).

CHARACTERISTICS	N	All women in the DiGest trial	N	Women with at least 24 hours of CGM data at any timepoint
Maternal age years	425	33.0 (5.0)	379	33.3 (4.9)
BMI at enrolment kg/m^2^	425	35.7 (6.4)	379	35.3 (6.2)
Ethnicity	425		379	
White		332 (78.1)		295 (77.4)
Asian		73 (17.2)		66 (17.4)
Black		17 (4.0)		17 (4.5)
Other		3 (0.7)		3 (0.8)
Primiparous	385	136 (35.3)	379	129 (36.7)
Maternal education (degree level or above)	425	201 (47.3)	379	191 (50.4)
Deprivation (IMD deciles)	425	6.5 (2.5)	379	6.6 (2.5)
Previous GDM	424	122 (28.8)	378	107 (28.3)
Gestational age at GDM diagnosis	414	22.9 (6.4)	369	22.8 (6.5)
GDM diagnosis before 28^th ^weeks	414	320 (77.3)	369	286 (77.5)
**GDM diagnosis before 20^th^ weeks**	414	118 (28.5)	369	106 (28.7)
Diagnosis method	425		379	
OGTT		217 (51.1)		192 (50.7)
HbA1c		98 (23.1)		90 (23.8)
Capillary glucose test		110 (25.9)		97 (25.6)
**GLYCEMIA LEVELS AND TREATMENT AT ENROLMENT**				
OGTT 0 hr glucose mmol/L	206	5.0 (0.7)	180	5.0 (0.7)
OGTT 0 hr glucose mg/dL	206	90.0 (12.6)	180	90.0 (12.6)
OGTT 2 hr glucose mmol/L	207	8.1 (1.7)	182	8.1 (1.6)
OGTT 2 hr glucose mg/dL	206	145.8 (31.0)	182	145.8 (29.0)
HbA1c mmol/mol	147	39.0 (4.6)	130	39.3 (4.6)
Medications at enrolment	425		379	
Metformin		94 (22.1)		83 (21.9)
Short-acting insulin		38 (8.9)		35 (9.2)
Long-acting insulin		101 (23.8)		95 (25.1)
**SUBOPTIMAL PREGNANCY OUTCOMES**				
**Medications at 36 weeks**				
**Metformin**	310	88 (28.4)	277	78 (28.2)
**Short-acting insulin**	310	41 (13.2)	277	91 (13.0)
**Long-acting insulin**	311	104 (33.4)	277	36 (32.9)
Preeclampsia	383	7 (1.8)	351	6/351 (1.7)
Preterm birth	384	36 (9.4)	352	31 (8.8)
Caesarean delivery	425	182(42.5)	379	166 (43.8)
LGA Intergrowth	382	74 (19.3)	350	63 (18.0)
SGA Intergrowth	382	17 (4.4)	350	16 (4.6)
NICU admission	382	40 (10.4)	350	31 (8.9)
Neonatal Hypoglycemia	382	19 (5.0)	350	17 (4.9)
Postnatal prediabetes	249	24 (9.6)	236	24 (10.2)
**CGM METRICS AT 29 WEEKS**			**Women with at least 24 hours of CGM data at 29 weeks**	
Days of CGM use			361	5.8 (2.2)
Mean CGM glucose mmol/L			361	5.8 (0.8)
Mean CGM glucose mg/dL			361	104.4 (14.4)
TIRp %			361	90.8 (11.0)
TARp %			361	7.5 (11.3)
Stringent TIRp %			361	77.0 (18.4)
Stringent TARp %			361	21.3 (19.2)
TBRp %			361	1.7 (2.9)
Nocturnal mean CGM glucose mmol/L			361	5.7 (0.9)
Nocturnal mean CGM glucose mg/dL			361	102.6 (16.2)
Nocturnal TIRp %			361	92.0 (12.7)
Nocturnal TARp %			361	5.7 (12.8)
Stringent nocturnal TIRp %			361	79.5 (23.0)
Stringent nocturnal TARp %			361	18.3 (23.8)
Nocturnal TBRp %			361	2.3 (4.3)
CV			361	18.2 (3.8)
SD			361	1.1 (0.3)
Mean CGM glucose <110mg/dL; 6.1mmol/L			361	268 (74.2)
TIR ≥90% target; 63-140 mg/dL; 3.5-7.8 mmol/L			361	263 (72.9)
TAR <10% target; 63-140 mg/dL; 3.5-7.8 mmol/l			361	279 (77.3)
TIR ≥70% target; 63-120 mg/dL; 3.5-6.7 mmol/L			361	275 (76.2)
TAR <30% target; 63-120 mg/dL; 3.5-6.7 mmol/L			361	276 (76.5)
TBR <4% target; 63 mg/dL; 3.5 mmol/L			361	307 (85.1)
Nocturnal* mean CGM glucose < 110mg/dL; 6.1mmol/L			361	261 (72.5)
Nocturnal TIR ≥90% target; 63-140 mg/dL; 3.5-7.8 mmol/L			361	277 (76.9)
Nocturnal TAR <10% target; 63-140 mg/dL; 3.5-7.8 mmol/l			361	302 (83.9)
Nocturnal TIR ≥70% target; 63-120 mg/dL; 3.5-7.8 mmol/L			361	274 (75.9)
Nocturnal TAR <30% target; 63-120 mg/dL; 3.5-7.8 mmol/l			361	276 (76.7)
Nocturnal TBR <4% target; 63 mg/dL; 3.5 mmol/L			361	297 (82.5)

* nocturnal CGM reading was missing for one participant.

BMI: body mass index; CGM: continuous glucose monitoring; CV: Coefficient of variation; GDM: gestational diabetes mellites; LGA: large for gestational age; OGTT: oral glucose tolerance test; SD: standard deviation; SGA; small for gestational age; TIRp: time in 63-140 mg/dL; 3.5-7.8 mmol/L; TARp: time above range 63-140 mg/dL; 3.5-7.8 mmol/L; TBRp: time below range 63-140 mg/dL; 3.5-7.8 mmol/l; TIRp stringent: time in range 63-120 mg/dL; 3.5-6.7 mmol/l time in range; TARp stringent: time above range 63-120 mg/dL; 3.5-6.7 mmol/L.

**Table 2:** Associations between CGM metrics at enrolment and pregnancy outcomes in women with gestational diabetes. Results are presented as odds ratio (95% confidence intervals). For the continuous CGM metrics the ORs are per standard deviation change in the metrics. Significance levels; *P<0.05; **P<0.01, ***P<0.001.

CGM METRICS AT ENROLMENT (~29 WEEKS); n=332^†^	LGA Intergrowth	SGA Intergrowth	Preterm	NICU admission	Neonatal Hypoglycemia
	n=61	n=16	n=30	n=30	n=17
**z-Mean CGM glucose mmol**	1.57 (1.16 to 2.13)**	1.89 (1.10 to 3.24)*	1.47 (0.99 to 2.18)	1.39 (0.93 to 2.10)	1.40 (0.87 to 2.24)
**z-TIR 63-140 mg/dL; 3.5-7.8 mmol/L**	0.54 (0.40 to 0.73)***	0.69 (0.44 to 1.09)	0.74 (0.52 to 1.04)	0.76 (0.54 to 1.07)	0.73 (0.49 to 1.08)
**z-TAR 63-140 mg/dL; 3.5-7.8 mmol/L**	1.80 (1.34 to 2.41)***	1.52 (0.97 to 2.37)	1.39 (0.99 to 1.95)	1.35 (0.96 to 1.89)	1.36 (0.92 to 2.01)
**z-TIR 63-120 mg/dL; 3.5-6.7 mmol/L**	0.66 (0.50 to 0.87)**	0.54 (0.33 to 0.89)*	0.78 (0.54 to 1.12)	0.73 (0.50 to 1.07)	0.70 (0.44 to 1.09)
**z-TAR 63-120 mg/dL; 3.5-6.7 mmol/L**	1.49 (1.13 to 1.97)**	1.88 (1.14 to 3.09)*	1.31 (0.90 to 1.89)	1.38 (0.94 to 2.01)	1.42 (0.90 to 2.23)
**z-TBR 63mg/dl; 3.5 mmol/L**	1.05 (0.77 to 1.43)	0.76 (0.39 to 1.48)	0.83 (0.50 to 1.36)	0.75 (0.39 to 1.44)	1.02 (0.59 to 1.75)
**z-Nocturnal mean 63-140** **mg/dL; 3.5-7.8 mmol/L**	1.56 (1.13 to 2.14)**	1.77 (1.04 to 2.99)*	1.35 (0.89 to 2.04)	1.15 (0.75 to 1.77)	1.23 (0.76 to 1.97)
**z-Nocturnal TIR 63-140** **mg/dL; 3.5-7.8 mmol/L**	0.62 (0.46 to 0.84)**	0.83 (0.50 to 1.38)	0.81 (0.56 to 1.18)	0.82 (0.57 to 1.17)	0.86 (0.60 to 1.25)
**z-Nocturnal TAR 63-140** **mg/dL; 3.5-7.8 mmol/L**	1.68 (1.24 to 2.29)**	1.33 (0.84 to 2.11)	1.25 (0.86 to 1.80)	1.22 (0.86 to 1.73)	1.15 (0.79 to 1.66)
**z-Nocturnal TIR 63-120** **mg/dL; 3.5-6.7 mmol/L**	0.72 (0.55 to 0.95)*	0.58 (0.35 to 0.96)*	0.87 (0.59 to 1.29)	0.84 (0.57 to 1.24)	0.78 (0.49 to 1.23)
**z-Nocturnal TAR 63-120** **mg/dL; 3.5-6.7 mmol/L**	1.41 (1.07 to 1.86)*	1.79 (1.09 to 2.94)*	1.15 (0.78 to 1.70)	1.19 (0.80 to 1.75)	1.27 (0.80 to 2.02)
**z-Nocturnal TBR 63 mg/dL;** **3.5 mmol/L**	0.78 (0.52 to 1.19)	0.67 (0.32 to 1.40)	0.97 (0.66 to 1.44)	0.98 (0.63 to 1.53)	1.05 (0.61 to 1.82)
**CV**	1.10 (1.02 to 1.19)*	1.03 (0.90 to 1.17)	1.04 (0.94 to 1.15)	1.02 (0.91 to 1.13)	0.99 (0.86 to 1.14)
**SD**	7.17 (2.53 to 20.32)***	3.30 (0.59 to 18.38)	3.10 (0.83 to 11.58)	2.21 (0.57 to 8.55)	2.16 (0.41 to 11.24)
**z-HbA1c (N=130)**	0.81 (0.49 to 1.32)	0.64 (0.12 to 3.27)	3.07 (1.43 to 6.58)**	0.35 (0.12 to 1.05)	0.39 (0.09 to 1.71)
**CGM TARGETS AT 29 WEEKS**					
**Mean CGM glucose <** **110mg/dL; 6.1mmol/L**	0.41 (0.22 to 0.77)**	0.26 (0.08 to 0.79)*	0.52 (0.23 to 1.19)	0.50 (0.21 to 1.18)	0.44 (0.15 to 1.29)
**TIR ≥90% target; 63-140** **mg/dL; 3.5-7.8 mmol/L**	0.38 (0.20 to 0.70)**	0.30 (0.10 to 0.91)*	0.50 (0.22 to 1.13)	0.47 (0.20 to 1.08)	0.43 (0.15 to 1.26)
**TIR ≥94% target; 63-140** **mg/dL; 3.5-7.8 mmol/L**	0.67 (0.37 to 1.20)	0.40 (0.13 to 1.19)	0.71 (0.33 to 1.54)	0.84 (0.37 to 1.89)	0.73 (0.25 to 2.11)
**TAR <10% target; 63-140** **mg/dL; 3.5-7.8 mmol/L**	0.39 (0.20 to 0.73)**	0.19 (0.06 to 0.62)**	0.40 (0.17 to 0.94)*	0.57 (0.23 to 1.38)	0.37 (0.13 to 1.09)
**TAR <4% target; 63-140** **mg/dL; 3.5-7.8 mmol/L**	0.59 (0.32 to 1.07)	0.31 (0.10 to 0.94)*	0.67 (0.30 to 1.47)	0.66 (0.28 to 1.51)	0.87 (0.30 to 2.51)
**TIR ≥70% target; 63-120** **mg/dL; 3.5-6.7 mmol/L**	0.38 (0.20 to 0.71)**	0.29 (0.09 to 0.94)*	0.53 (0.23 to 1.25)	0.59 (0.24 to 1.41)	0.40 (0.14 to 1.19)
**TAR <30% target; 63-120** **mg/dL; 3.5-6.7 mmol/L**	0.37 (0.20 to (0.69)**	0.29 (0.09 to 0.92)*	0.52 (0.22 to 1.22)	0.52 (0.22 to 1.19)	0.39 (0.13 to 1.16)
**TBR <4% target; 63 mg/dL;** **3.5 mmol/L**	0.96 (0.39 to 2.35)	1.53 (0.32 to 7.25)	0.92 (0.32 to 2.61)	1.54 (0.41 to 5.80)	0.56 (0.13 to 2.30)
**Mean nocturnal glucose <** **110mg/dL; 6.1mmol/L**	0.34 (0.18 to 0.63)**	0.17 (0.05 to 0.57)	0.42 (0.19 to 0.97)*	0.49 (0.21 to 1.12)	0.50 (0.17 to 1.45)
**Nocturnal TIR ≥90% target;** **63-140 mg/dL; 3.5-7.8 mmol/L**	0.43 (0.22 to 0.82)*	0.75 (0.23 to 2.50)	0.76 (0.32 to 1.82)	0.73 (0.30 to 1.75)	0.55 (0.18 to 1.68)
**Nocturnal TAR <10% target;** **63-140 mg/dL; 3.5-7.8 mmol/L**	0.34 (0.17 to 0.68)**	0.51 (0.13 to 2.02)	0.83 (0.29 to 2.35)	0.53 (0.21 to 1.36)	0.50 (0.15 to 1.65)
**Nocturnal TIR ≥70% target;** **63-120 mg/dL; 3.5-6.7 mmol/L**	0.47 (0.25 to 0.88)*	0.26 (0.08 to 0.86)*	0.76 (0.31 to 1.83)	0.77 (0.31 to 1.92)	0.74 (0.24 to 2.32)
**Nocturnal TAR <30% target;** **63-120 mg/dL; 3.5-6.7 mmol/L**	0.45 (0.24 to 0.86)*	0.25 (0.07 to 0.82)*	0.71 (0.29 to 1.73)	0.75 (0.30 to 1.87)	0.70 (0.22 to 2.20)
**Nocturnal TBR <4% target;** **63 mg/dL; 3.5 mmol/L**	1.60 (0.63 to 4.09)	1.19 (0.31 to 4.56)	0.96 (0.36 to 2.55)	1.17 (0.41 to 3.37)	1.28 (0.26 to 6.32)
**CGM MARKERS OF INCREASED RISK AT 29 WEEKS**					
**Mean CGM glucose ≥** **110mg/dL; 6.1mmol/L**	2.44 (1.31 to 4.53)**	3.91 (1.27 to 12.08)*	1.92 (0.84 to 4.39)	2.11 (0.90 to 4.93)	2.20 (0.75 to 6.44)
**TIR <90% target; 63-140** ** mg/dL; 3.5-7.8 mmol/L**	2.66 (1.43 to 4.96)**	3.31 (1.09 to 10.00)*	1.99 (0.88 to 4.51)	2.02 (0.86 to 4.74)	2.26 (0.78 to 6.60)
**TIR <94% target; 63-140** **mg/dL; 3.5-7.8 mmol/L**	1.50 (0.83 to 2.70)	2.52 (0.84 to 7.54)	1.40 (0.65 to 3.04)	1.19 (0.53 to 2.69)	1.37 (0.47 to 3.98)
**TAR ≥10% target; 63-140** **mg/dL; 3.5-7.8 mmol/L**	2.60 (1.37 to 4.95)**	5.18 (1.61 to 16.63)**	2.48 (1.07 to 5.77)*	1.92 (0.80 to 4.59)	2.76 (0.94 to 8.14)
**TAR ≥4% target; 63-140** **mg/dL; 3.5-7.8 mmol/L**	1.70 (0.94 to 3.10)	3.24 (1.06 to 9.87)*	1.50 (0.68 to 3.29)	1.53 (0.66 to 3.53)	1.15 (0.40 to 3.32)
**TIR <70% target; 63-120** **mg/dL; 3.5-6.7 mmol/L**	2.65 (1.41 to 4.98)**	3.41 (1.06 to 10.98)*	1.88 (0.80 to 4.39)	1.70 (0.71 to 4.09)	2.55 (0.86 to 7.52)
**TAR ≥30% target; 63-120** **mg/dL; 3.5-6.7 mmol/L**	2.71 (1.44 to 5.09)**	3.50 (1.09 to 11.30)*	1.92 (0.82 to 4.50)	1.79 (0.75 to 4.29)	2.50 (0.84 to 7.43)
**TBR ≥4% target; 63 mg/dL;** **3.5 mmol/l**	1.04 (0.43 to 2.56)	0.65 (0.14 to 3.10)	1.09 (0.38 to 3.11)	0.68 (0.18 to 2.55)	1.84 (0.45 to 7.55)
**Mean nocturnal glucose ≥** **110mg/dL; 6.1mmol/L**	2.97 (1.58 to 5.58)**	5.74 (1.75 to 18.84)	2.36 (1.04 to 5.36)*	2.05 (0.90 to 4.67)	2.00 (0.69 to 5.84)
**Nocturnal TIR <90% target;** **63-140 mg/dL; 3.5-7.8 mmol/L**	2.33 (1.22 to 4.44)*	1.33 (0.40 to 4.44)	1.31 (0.55 to 3.13)	1.38 (0.57 to 3.32)	1.82 (0.60 to 5.59)
**Nocturnal TAR ≥10% target;** **63-140 mg/dL; 3.5-7.8 mmol/L**	2.99 (1.48 to 6.03)**	1.97 (0.50 to 7.86)	1.21 (0.42 to 3.43)	1.89 (0.73 to 4.86)	1.99 (0.61 to 6.53)
**Nocturnal TIR <70% target;** **63-120 mg/dL; 3.5-6.7 mmol/L**	2.14 (1.13 to 4.05)*	3.83 (1.16 to 12.66)*	1.32 (0.55 to 3.21)	1.30 (0.52 to 3.24)	1.35 (0.43 to 4.20)
**Nocturnal TAR ≥30% target;** **63-120 mg/dL; 3.5-6.7 mmol/L**	2.22 (1.17 to 4.20)*	4.07 (1.22 to 13.58)*	1.41 (0.58 to 3.42)	1.34 (0.53 to 3.35)	1.43 (0.46 to 4.49)
**Nocturnal TBR ≥4% target;** **63 mg/dL; 3.5 mmol/l**	0.62 (0.24 to 1.59)	0.84 (0.22 to 3.21)	1.04 (0.39 to 2.78)	0.85 (0.30 to 2.46)	0.78 (0.16 to 3.83)

† The number of women with data for all variables entered in the logistic regression model CGM: continuous glucose monitoring; LGA: large for gestational age; SGA: small for gestational age; NICU: neonatal intensive care unit; TIR: time in range; TAR: time above range; TBR: time below range; CV: Coefficient of variation; SD: standard deviation.

**Table 3:** ROC analysis and optimal CGM thresholds at 29 weeks of gestation for pregnancy outcome prediction. Results are presented as area under the ROC analysis (95% confidence intervals). Significance levels; *P<0.05; **P<0.01, ***P<0.001.

	LGA Intergrowth	SGA Intergrowth	Preterm	NICU admission	Neonatal Hypoglycemia
**Mean CGM glucose mmol/L**					
ROC results, AUC (95%CI)	0.61 (0.52 to 0.69)*	0.62 (0.48 to 0.76)	0.59 (0.48 to 0.70)	0.58 (0.46 to 0.70)	0.57 (0.38 to 0.75)
Optimal threshold for sens & spec	110 mg/dL (6.1mmol/L)	103 mg/dL (5.7mmol/L)	103 mg/dL (5.7mmol/L)	115 mg/dL (6.4mmol/L)	120 mg/dL (6.7mmol/L)
Sensitivity at threshold	0.54	0.69	0.61	0.53	0.47
Specificity at threshold	0.64	0.57	0.47	0.63	0.88
**TIR 63-140 mg/dL; 3.5-7.8 mmol/L**					
ROC results, AUC (95%CI)	0.62 (0.53 to 0.71)**	0.62 (0.48 to 0.76)	0.59 (0.49 to 0.70)	0.59 (0.48 to 0.71)	0.61 (0.45 to 0.78)
Optimal threshold for sens & spec	93.5	90.7	94.5	95.6	94.8
Sensitivity at threshold	0.56	0.56	0.63	0.73	0.71
Specificity at threshold	0.6	0.7	0.61	42	0.48
**TAR 63-140 mg/dL; 3.5-7.8 mmol/L**					
ROC results, AUC (95%CI)	0.61 (0.52 to 0.70)*	0.62 (0.48 to 0.76)	0.59 (0.48 to 0.70)	0.61 (0.51 to 0.72)*	0.56 (0.38 to 0.74)
Optimal threshold for sens & spec	3.5	10.6	2.2	3.6	15.1
Sensitivity at threshold	0.64	0.44	0.77	0.64	0.47
Specificity at threshold	0.56	0.8	0.39	0.57	0.86
**TIR 63-120 mg/dL; 3.5-6.7 mmol/L**					
ROC results, AUC (95%CI)	0.61 (0.52 to 0.69)*	0.61 (0.47 to 0.75)	0.58 (0.47 to 0.68)	0.59 (0.48 to 0.71)	0.55 (0.36 to 0.73)
Optimal threshold for sens & spec	80.1	74.2	81.1	84.9	58.1
Sensitivity at threshold	0.56	0.56	0.57	0.77	0.47
Specificity at threshold	0.6	0.72	0.56	0.44	0.86
**TAR 63-120 mg/dL; 3.5-6.7 mmol/L**					
ROC results, AUC (95%CI)	0.59 (0.51 to 0.68)*	0.61 (0.47 to 0.75)	0.57 (0.47 to 0.68)	0.59 (0.48 to 0.70)	0.54 (0.36 to 0.73)
Optimal threshold for sens & spec	16.2	23.9	12.8	13.3	41.8
Sensitivity at threshold	0.61	0.56	0.73	0.73	0.47
Specificity at threshold	0.55	0.72	0.42	0.44	0.86
**Nocturnal mean glucose mmol/L**					
ROC results, AUC (95%CI)	0.61 (0.53 to 0.69)**	0.63 (0.49 to 0.77)	0.56 (0.45 to 0.67)	0.54 (0.42 to 0.66)	0.56 (0.39 to 0.73)
Optimal threshold for sens & spec	108mg/dL (6mmol/L)	110 mg/dL (6.1mmol/L)	106mg/dL (5.9mmol/L)	112mg/dL (6.2mmol/L)	110 mg/dL (6.1mmol/L)
Sensitivity at threshold	0.52	0.56	0.47	0.4	0.47
Specificity at threshold	0.75	0.73	0.69	0.77	0.75
**Nocturnal TIR 63-140 mg/dL; 3.5-7.8 mmol/L**					
ROC results, AUC (95%CI)	0.59 (0.50 to 0.67)*	0.60 (0.48 to 0.72)	0.57 (0.47 to 0.67)	0.54 (0.43 to 0.66)	0.56 (0.42 to 0.71)
Optimal threshold for sens & spec	92.2	97.7	99.4	81.9	83.6
Sensitivity at threshold	0.44	0.88	0.97	0.27	0.35
Specificity at threshold	0.74	0.41	0.21	0.9	0.88
**Nocturnal TAR 63-140 mg/dL; 3.5-7.8 mmol/L**					
ROC results, AUC (95%CI)	0.64 (0.56 to 0.72)**	0.65 (0.53 to 0.77)*	0.58 (0.47 to 0.68)	0.52 (0.41 to 0.64)	0.55 (0.41 to 0.70)
Optimal threshold for sens & spec	5.7	0.3	0.2	19.8	16.3
Sensitivity at threshold	0.43	0.81	0.67	0.2	0.29
Specificity at threshold	0.81	0.53	0.52	0.93	0.89

CGM: continuous glucose monitoring; LGA: large for gestational age; NICU: neonatal intensive care unit; ROC; receiver operating characteristic; sens & spec; sensitivity and specificity TIR: time in range; TAR: time above range; TBR: time below range;

## Article highlights

- Why did we undertake this study?
  CGM is increasingly used in gestational diabetes but the optimal metrics and targets in this population are undefined.
- What is the question we wanted to answer?
  Which CGM metrics and thresholds could be used to predict outcomes in GDM?
- What did we find?
  CGM metrics at 29, 32 and 36 weeks were associated with pregnancy outcomes. Maintaining glycemia within optimal targets of mean glucose <110mg/dL (6.1mmol/L), TIRp ≥90% or TARp <10% was associated with reduced risk of LGA and SGA.
- What are the implications of our findings?
  In women with GDM, CGM mean glucose <110mg/dL (6.1mmol/L), ≥90% TIRp or <10% TARp using a range of 63-140mg/dL (3.5-7.8mmol/L) at 29 weeks of gestation was associated with a low risk of suboptimal offspring outcomes.

## References

[1] Wang H, Li N, Chivese T, Werfalli M, Sun H, Yuen L (2022). IDF Diabetes Atlas: Estimation of Global and Regional Gestational Diabetes Mellitus Prevalence for 2021 by International Association of Diabetes in Pregnancy Study Group’s Criteria. Diabetes Res Clin Pract.

[2] Meek CL (2023). An unwelcome inheritance: childhood obesity after diabetes in pregnancy. Diabetologia.

[3] Liang X, Fu Y, Lu S, Shuai M, Miao Z, Gou W (2023). Continuous glucose monitoring-derived glycemic metrics and adverse pregnancy outcomes among women with gestational diabetes: a prospective cohort study. Lancet Reg Heal West Pacific.

[4] Saravanan P, Magee LA, Banerjee A, Coleman MA, Von Dadelszen P, Denison F (2020). Gestational diabetes: opportunities for improving maternal and child health. Lancet Diabetes Endocrinol.

[5] Feig DS, Donovan LE, Corcoy R, Murphy KE, Amiel SA, Hunt KF (2017). Continuous glucose monitoring in pregnant women with type 1 diabetes (CONCEPTT): a multicentre international randomised controlled trial. Lancet.

[6] Battelino T, Danne T, Bergenstal RM, Amiel SA, Beck R, Biester T (2019). Clinical targets for continuous glucose monitoring data interpretation: Recommendations from the international consensus on time in range. Diabetes Care.

[7] Battelino T, Alexander CM, Amiel SA, Arreaza-Rubin G, Beck RW, Bergenstal RM (2023). Continuous glucose monitoring and metrics for clinical trials: an international consensus statement. Lancet Diabetes Endocrinol.

[8] Carlson AL, Beck RW, Li Z, Norton E, Bergenstal RM, Johnson M (2024). Glucose levels measured with continuous glucose monitoring in uncomplicated pregnancies. BMJ Open Diabetes Res Care.

[9] Law GR, Alnaji A, Alrefaii L, Endersby D, Cartland SJ, Gilbey SG (2019). Suboptimal Nocturnal Glucose Control Is Associated With Large for Gestational Age in Treated Gestational Diabetes Mellitus. Diabetes Care.

[10] Kusinski LC, Murphy HR, De Lucia Rolfe E, Rennie KL, Oude Griep LM, Hughes D (2020). Dietary Intervention in Pregnant Women with Gestational Diabetes; Protocol for the DiGest Randomised Controlled Trial. Nutrients.

[11] Kusinski LC, Jones D, Atta N, Turner E, Smith S, Oude Griep LM (2025). Reduced-energy diet in women with gestational diabetes: the dietary intervention in gestational diabetes DiGest randomized clinical trial. Nat Med.

[12] Royal College of Obstetricians and Gynaecologists (2020). Information for healthcare professionals Guidance for maternal medicine services in the coronavirus (COVID-19) pandemic Summary of updates.

[13] National Institute for Health and Care Excellence (2015). Diabetes in pregnancy : management from preconception to the postnatal period. NICe.

[14] Royal College of Obstetricians and Gynaecologists (2020). Guidance for maternal medicine services in the coronavirus (COVID-19) pandemic.

[15] Egan AM, Bogdanet D, Griffin TP, Kgosidialwa O, Cervar-Zivkovic M, Dempsey E (2020). A core outcome set for studies of gestational diabetes mellitus prevention and treatment. Diabetologia.

[16] Overview | Hypertension in pregnancy: diagnosis and management | Guidance.

[17] Scott EM, Murphy HR, Myers J, Saravanan P, Poston L, Law GR (2023). MAGIC (maternal glucose in pregnancy) understanding the glycemic profile of pregnancy, intensive CGM glucose profiling and its relationship to fetal growth: an observational study protocol. BMC Pregnancy Childbirth.

[18] Scott EM, Feig DS, Murphy HR, Law GR (2020). Continuous Glucose Monitoring in Pregnancy: Importance of Analyzing Temporal Profiles to Understand Clinical Outcomes. Diabetes Care.

[19] de Hond AAH, Steyerberg EW, van Calster B (2022). The Lancet Digital Health.

[20] Liu X (2012). Classification accuracy and cut pointselection. Stat Med.

[21] Sarno L, Savu A, Adam S, Masulli M, Wu N, Yang Y (2022). Gestational Diabetes Mellitus and Preeclampsia: Correlation and Influencing Factors. Front Cardiovasc Med.

[22] Malaza N, Masete M, Adam S, Dias S, Nyawo T, Pheiffer C (2022). A Systematic Review to Compare Adverse Pregnancy Outcomes in Women with Pregestational Diabetes and Gestational Diabetes. Int J Environ Res Public Health.

[23] Durnwald C, Beck RW, Li Z, Norton E, Bergenstal RM, Johnson M (2024). Continuous Glucose Monitoring Profiles in Pregnancies With and Without Gestational Diabetes Mellitus. Diabetes Care.

[24] Li Z, Beck R, Durnwald C, Carlson A, Norton E, Bergenstal R (2024). Continuous Glucose Monitoring Prediction of Gestational Diabetes Mellitus and Perinatal Complications. Diabetes Technol Ther.

[25] Meek CL, Tundidor D, Feig DS, Yamamoto JM, Scott EM, Ma DD (2021). Novel Biochemical Markers of Glycemia to Predict Pregnancy Outcomes in Women With Type 1 Diabetes. Diabetes Care.

[26] Sanusi AA, Xue Y, McIlwraith C, Howard H, Brocato BE, Casey B (2024). Association of Continuous Glucose Monitoring Metrics With Pregnancy Outcomes in Patients With Preexisting Diabetes. Diabetes Care.

[27] Durnwald C, Beck RW, Li Z, Norton E, Bergenstal R, Johnson M (2024). Continuous Glucose Monitoring–Derived Differences in Pregnancies With and Without Adverse Perinatal Outcomes. Obstet Gynecol.

[28] Ornoy A, Becker M, Weinstein-Fudim L, Ergaz Z (2021). Diabetes during Pregnancy: A Maternal Disease Complicating the Course of Pregnancy with Long-Term Deleterious Effects on the Offspring. A Clinical Review. Int J Mol Sci.

[29] Li J, Pan Y, Zheng Q, Chen X, Jiang X, Liu R (2024). Risk factors and glycaemic control in small-for-gestational-age infants born to mothers with gestational diabetes mellitus: a case–control study using propensity score matching based on a large population. BMJ Open.

[30] Levy CJ, Galindo RJ, Parkin CG, Gillis J, Argento NB (2024). All Children Deserve to Be Safe, Mothers Too: Evidence and Rationale Supporting Continuous Glucose Monitoring Use in Gestational Diabetes Within the Medicaid Population. J Diabetes Sci Technol.

[31] Wyckoff JA, Brown FM (2021). Time in Range in Pregnancy: Is There a Role?. Diabetes Spectr.

[32] Polonsky WH, Hessler D, Group for the DS, Hessler D, Group for the DS, Ruedy KJ, Group for the DS (2017). The Impact of Continuous Glucose Monitoring on Markers of Quality of Life in Adults With Type 1 Diabetes: Further Findings From the DIAMOND Randomized Clinical Trial. Diabetes Care.

[33] Yoo JH, Kim JH (2023). The Benefits Of Continuous Glucose Monitoring In Pregnancy. Endocrinol Metab.

[34] Kusinski LC, Brown J, Hughes DJ, Meek CL (2023). Feasibility and acceptability of continuous glucose monitoring in pregnancy for the diagnosis of gestational diabetes: A single-centre prospective mixed methods study. PLoS One.

