# Supplementary material for: Continuous glucose monitoring metrics and pregnancy outcomes in women with gestational diabetes: a secondary analysis of the DiGest trial
Source: Diabetes Care. Author manuscript; Available in PMC 2026 Mar 4. (PMC7618813; doi:10.2337/dc25-0452)
Supplement: Supplementary Tables and Figures [file EMS211987-supplement-Supplementary_Tables_and_Figures.pdf]

SUPPLEMENTARY MATERIAL

Tables

Supplementary Table 1. Detailes of the study search strategy .

Supplementary Table 2. Checklist of items to include when reporting a systematic review or meta-analysis- PRISMA.

Supplementary Table 3. Details of heterogeneity measures in the meta-analysis.

Figures

Supplementary Figure 1. Forest plot of subgroup analysis – BMI (categorical and continuous variables)

Supplementary Figure 2. Forest plot of subgroup analysis – GWG (categorical and continuous variables)

Supplementary Figure 3a and 3b. Bubble plot of the association between BMI and perinatal outcomes: (a) the role of the publication year; (b) the role of the sample size on the heterogeneity of the results

Supplementary Figure 4. Bubble plot of the association between GWG and perinatal outcomes: the role of the number of cases on the heterogeneity of the results

Supplementary Figure 5a and 5b. Funnel plots of the studies included in the meta-analysis, (a) BMI and (b) GWG

Supplementary Figure 6. Risk of bias appraisal in the primary studies using the CASP tool

Supplementary References: Reference list of the studies included in the systemtic review and meta analysis

Supplementary Table 1. Detailes of the study search strategy. The updated search was conducted on 16 January 2024.

A) Eligibility Criteria

PI(E)COS	Inclusion Criteria	Exclusion Criteria
Population	• Diagnosis of Type 1 diabetes<br>• Any stage of pregnancy	• Other types of diabetes<br>• Animal studies, cell work
Intervention/exposure	• Abnormal BMI (based on maternal BMI measured immediately befor or after conception)<br>• Abnormal GWG (based on weight change between the first weight measured around the time of conception and the latest recorded weight before delivery.)	• Unable to record maternal BMI and/or GWG
Comparison	• Optimal BMI (based on maternal BMI measured immediately befor or after conception)<br>• Adequate GWG (based on weight change between the first weight measured around the time of conception and the latest recorded weight before delivery.)	• Unable to record BMI and/or GWG
Outcomes	• Suboptimal pregnancy outcomes:<br>• Maternal outcomes: preeclampsia, preterm labour, perineal tears<br>• Neonatal outcomes: including but not limited to congenital anomalies, LGA, SGA, macrosomia, NICU admission, neonatal hypoglycaemia, jaundice, preterm birth, perinatal death, low APGAR score<br>• Obstetric outcomes: Caesarean section (mode of delivery), shoulder dystocia,	• No objectively measured perinatal outcomes of pregnancy
Study design	• Any study with analytical design<br>• No restriction on publication date<br>• Texts written in English only	• Descriptive studies

B) A list of the keywords used for databases search

((pregnant women OR pregnancies OR pregnancy) AND (type 1 diabetes OR diabetes type 1 OR diabetes type one OR type one diabetes OR T1D)) AND ((gestational weight gain OR pregnancy weight gain OR weight gain in pregnancy OR excess weight gain in pregnancy) OR (Pre-pregnancy BMI OR first trimester BMI OR Body Mass index) OR (Obese OR Obesity)) AND (pregnancy outcome OR adverse outcomes of pregnancy OR maternal complications OR neonatal complications OR delivery complications OR pregnancy complications OR labour complications OR labor complications)

C) An example of the databeses search Database:

Embase <1974 to 2024 January 16>
Ovid MEDLINE(R) and Epub Ahead of Print, In-Process, In-Data-Review & Other Non-Indexed Citations

#	Query	Results from 16 Jan 2024
1	Diabetes Mellitus, Type 1/	80,077
2	Body Mass Index/ or Gestational Weight Gain/	631,746
3	pregnancy outcomes.mp. or Pregnancy Outcome/	110,981
4	pregnancy weight gain.ti,ab.	1,162
5	type 1 diabetes.ti,ab.	102,551
6	pregnancy complication.ti,ab.	2,116
7	type one diabetes.ti,ab.	259
8	weight gain in pregnancy.ti,ab.	715
9	excess weight gain in pregnancy.ti,ab.	30
10	adverse outcomes of pregnancy.ti,ab.	723
11	labor complications.ti,ab.	332
12	1 or 5 or 7	141,677
13	2 or 8 or 9	632,141
14	3 or 6 or 10 or 11	113,353
15	4 or 13	632,649
16	12 and 14 and 15	203
17	1 and 2 and 3	149
18	Obesity/	601,538
19	obese.ti,ab.	291,203
20	15 or 18 or 19	1,143,254
21	12 and 14 and 20	269

BMI: Body mass index; GWG: gestational weight gain; LGA: Large for gestational age; SGA: Small for gestational age, NICU: Neonatal intensive care unit; APGAR: Appearance, Pulse, Grimace, Activity, Respiration

Supplementary Table 2 - Checklist of items to include when reporting a systematic review or meta-analysis

Section/topic	#	Checklist item	Reported on page #
**TITLE**			
Title	1	Identify the report as a systematic review, meta-analysis, or both.	Title page
**ABSTRACT**			
Structured summary	2	Provide a structured summary including, as applicable: background; objectives; data sources; study eligibility criteria, participants, and interventions; study appraisal and synthesis methods; results; limitations; conclusions and implications of key findings; systematic review registration number.	Abstract
**INTRODUCTION**			
Rationale	3	Describe the rationale for the review in the context of what is already known.	Introduction paragraph 2-3
Objectives	4	Provide an explicit statement of questions being addressed with reference to participants, interventions, comparisons, outcomes, and study design (PICOS).	Introduction paragraph 4
**METHODS**			
Protocol and registration	5	Indicate if a review protocol exists, if and where it can be accessed (e.g., Web address), and, if available, provide registration information including registration number.	Methods paragraph 1
Eligibility criteria	6	Specify study characteristics (e.g., PICOS, length of follow-up) and report characteristics (e.g., years considered, language, publication status) used as criteria for eligibility, giving rationale.	Methods, Search strategy: paragraph 1, Eligibility criteria: paragraph 1-3 and Supplementary Table1
Information sources	7	Describe all information sources (e.g., databases with dates of coverage, contact with study authors to identify additional studies) in the search and date last searched.	Methods, Search strategy: paragraph 1
Search	8	Present full electronic search strategy for at least one database, including any limits used, such that it could be repeated.	Methods, Search strategy: paragraph 1 and Supplementary Table1 and Figure 1
Study selection	9	State the process for selecting studies (i.e., screening, eligibility, included in systematic review, and, if applicable, included in the meta-analysis).	Methods, Search strategy, paragraph 1-2 and Figure 1
Data collection process	10	Describe method of data extraction from reports (e.g., piloted forms, independently, in duplicate) and any processes for obtaining and confirming data from investigators.	Methods, Search strategy, paragraph 2 and Data extraction paragraph 1-3
Data items	11	List and define all variables for which data were sought (e.g., PICOS, funding sources) and any assumptions and simplifications made.	Methods, Data extraction: paragraph 1-3, data synthesis paragraphs 2-3 and Supplementary Table1

Section/topic	#	Checklist item	Reported on page #
Risk of bias in individual studies	12	Describe methods used for assessing risk of bias of individual studies (including specification of whether this was done at the study or outcome level), and how this information is to be used in any data synthesis.	Methods, Data extraction: paragraph 4 and Supplementary Figure 16
Summary measures	13	State the principal summary measures (e.g., risk ratio, difference in means).	Methods, Data extraction: paragraph 3, data synthesis paragraphs 4
Synthesis of results	14	Describe the methods of handling data and combining results of studies, if done, including measures of consistency (e.g., I^2) for each meta-analysis.	Methods, Data synthesis and analysis: paragraph 1-5
Risk of bias across studies	15	Specify any assessment of risk of bias that may affect the cumulative evidence (e.g., publication bias, selective reporting within studies).	Methods, Data synthesis and analysis: paragraph 5
Additional analyses	16	Describe methods of additional analyses (e.g., sensitivity or subgroup analyses, meta-regression), if done, indicating which were pre-specified.	Methods, Data synthesis and analysis: paragraph 4 and 5
RESULTS			
Study selection	17	Give numbers of studies screened, assessed for eligibility, and included in the review, with reasons for exclusions at each stage, ideally with a flow diagram.	Results, study selection and characteristics: paragraph 1-2 and Figure 1
Study characteristics	18	For each study, present characteristics for which data were extracted (e.g., study size, PICOS, follow-up period) and provide the citations.	Results, study selection and characteristics: paragraph 1-2 and Table 1
Risk of bias within studies	19	Present data on risk of bias of each study and, if available, any outcome-level assessment (see Item 12).	Results, Risk of bias: paragraph 1-3 and Supplementary Figure 6
Results of individual studies	20	For all outcomes considered (benefits or harms), present, for each study: (a) simple summary data for each intervention group and (b) effect estimates and confidence intervals, ideally with a forest plot.	Results, Table 1 and Figures 2- 3
Synthesis of results	21	Present results of each meta-analysis done, including confidence intervals and measures of consistency.	Results, Heterogeneity and publication bias, Figures 2- 3, Supplementary Table 3 and Supplementary Figures 1-2
Risk of bias across studies	22	Present results of any assessment of risk of bias across studies (see Item 15).	Results, Risk of bias: paragraph 4 and Supplementary Figure 6
Additional analysis	23	Give results of additional analyses, if done (e.g., sensitivity or subgroup analyses, meta-regression [see Item 16]).	Results, heterogeneity and publication bias and Supplementary Figures 1-5
DISCUSSION			
Summary of evidence	24	Summarize the main findings including the strength of evidence for each main outcome; consider their relevance to key groups (e.g., health care providers, users, and policy makers).	Discussion paragraph 1 and 4-8

Section/topic	#	Checklist item	Reported on page #
Limitations	25	Discuss limitations at study and outcome level (e.g., risk of bias), and at review level (e.g., incomplete retrieval of identified research, reporting bias).	Discussion, Strength and weaknesses: paragraph 1-4
Conclusions	26	Provide a general interpretation of the results in the context of other evidence, and implications for future research.	Discussion, paragraph 6-9
FUNDING			
Funding	27	Describe sources of funding for the systematic review and other support (e.g., supply of data); role of funders for the systematic review.	Funding and Assistance, paragraph 1

Supplementary Table 3: Details of heterogeneity measures in the meta-analysis

Periconception BMI

Heterogeneity Measures

Preeclampsia	Tau-squared	.000
	H-squared	1.000
	I-squared (%)	.0
Preterm birth	Tau-squared	.085
	H-squared	8.070
	I-squared (%)	87.6
Caesarean section	Tau-squared	.024
	H-squared	4.838
	I-squared (%)	79.3
LGA	Tau-squared	.003
	H-squared	2.229
	I-squared (%)	55.1
Macrosomia	Tau-squared	.035
	H-squared	1.682
	I-squared (%)	40.6
Hypertensive disorders	Tau-squared	.000
	H-squared	1.000
	I-squared (%)	.0
Perinatal death	Tau-squared	.005
	H-squared	1.363
	I-squared (%)	26.6
Cong.malformation	Tau-squared	.000
	H-squared	1.000
	I-squared (%)	.0
Neonatal hypoglycaemia	Tau-squared	.000
	H-squared	1.000
	I-squared (%)	.0
NICU admission	Tau-squared	.000
	H-squared	1.000
	I-squared (%)	.0
Neonatal jaundice	Tau-squared	.025
	H-squared	1.391
	I-squared (%)	28.1
Overall	Tau-squared	.006
	H-squared	2.899
	I-squared (%)	65.5

Gestational Weight Gain

Heterogeneity Measures

Preeclampsia	Tau-squared	.001
	H-squared	1.366
	I-squared (%)	26.8
Preterm birth	Tau-squared	.000
	H-squared	1.000
	I-squared (%)	.0
Caesarean section	Tau-squared	.000
	H-squared	1.000
	I-squared (%)	.0
LGA	Tau-squared	.060
	H-squared	1.787
	I-squared (%)	44.0
Macrosomia	Tau-squared	.066
	H-squared	1.652
	I-squared (%)	39.5
SGA	Tau-squared	.000
	H-squared	1.000
	I-squared (%)	.0
RDS	Tau-squared	.000
	H-squared	1.000
	I-squared (%)	.0
Neonatal hypoglycaemia	Tau-squared	.000
	H-squared	1.000
	I-squared (%)	.0
NICU admission	Tau-squared	.000
	H-squared	1.000
	I-squared (%)	.0
Neonatal jaundice	Tau-squared	.373
	H-squared	2.972
	I-squared (%)	66.4
Overall	Tau-squared	.051
	H-squared	3.437
	I-squared (%)	70.9

BMI: Body mass index;; LGA: Large for gestational age; SGA: Small for gestational age, RDS: Respiratory distress syndrome; NICU: Neonatal intensive care unit

BMI Variable	Study ID	OR	Lower	Upper	p-value	Weight	Weight (%)	N_cases
Categoric BMI≥25	Oppermann, 2020	1.29	0.46	3.55	0.63	3.63	0.14	30
	Kawakita, 2016	1.66	0.71	3.89	0.24	5.12	0.20	42
	Persson, 2016	1.24	1.09	1.43	0.00	89.78	3.43	972
	Meek, 2023	2.17	0.82	5.74	0.12	3.96	0.15	20
	Kawakita, 2016	0.52	0.25	1.09	0.08	6.77	0.26	95
	Meek, 2023	2.01	1.08	3.71	0.03	9.54	0.36	70
	Persson, 2012	1.29	1.12	1.49	0.00	86.65	3.31	741
	Stogianni, 2019	1.07	0.33	3.47	0.91	2.72	0.10	36
	Kawakita, 2016	0.74	0.42	1.31	0.30	11.14	0.43	204
	Meek, 2023	1.23	0.65	2.32	0.52	9.04	0.35	116
	Persson, 2012	1.51	1.24	1.84	0.00	60.04	2.29	1749
	Stogianni, 2019	2.48	0.74	8.27	0.14	2.60	0.10	22
	Meek, 2023	0.76	0.41	1.40	0.38	9.65	0.37	68
	Persson, 2012	1.13	1.01	1.27	0.04	103.59	3.95	1694
	Kawakita, 2016	1.15	0.63	2.12	0.65	9.71	0.37	71
	Cundy, 2002	1.80	0.81	4.00	0.15	5.81	0.22	45
	Persson, 2012	1.34	0.75	2.40	0.32	10.61	0.40	35
	Persson, 2012	1.27	0.69	2.33	0.44	9.81	0.37	150
	Meek, 2023	1.04	0.53	2.03	0.92	8.12	0.31	47
	Meek, 2023	0.95	0.51	1.78	0.88	9.15	0.35	59
	Meek, 2023	0.66	0.33	1.33	0.25	7.40	0.28	42
	Subgroup Overall	1.22	1.11	1.34	0.00			
Continuous	Abell, 2016	0.98	0.91	1.06	0.61	128.87	4.92	42
	Soholm, 2021	0.84	0.42	1.70	0.63	7.44	0.28	38
	Weschenfelder, 2021	1.02	0.53	1.97	0.96	8.35	0.32	51
	Abell, 2016	1.03	0.95	1.12	0.48	124.96	4.77	66
	Klemetti, 2012	1.02	0.98	1.07	0.36	148.75	5.68	677
	Lepercq, 2010	1.22	0.71	2.10	0.47	12.13	0.46	148
	McWhorter-PPG, 2018	1.38	0.93	2.06	0.11	21.08	0.80	134
	McWhorter-CSL, 2018	0.94	0.64	1.39	0.77	21.82	0.83	131
	Abell, 2016	1.06	0.98	1.14	0.14	128.87	4.92	47
	Morrens, 2016	0.99	0.92	1.06	0.78	132.72	5.06	117
	Scifres, 2014	1.10	1.04	1.16	0.00	141.25	5.39	114
	Ladfors, 2018	1.02	0.95	1.09	0.55	133.98	5.11	111
	Alexander, 2019	0.94	0.87	1.01	0.10	130.16	4.97	59
	Aschwald, 2009	1.13	1.02	1.25	0.01	114.43	4.37	40
	Klemetti, 2012	1.06	1.01	1.11	0.01	148.75	5.68	304
	Evers, 2002	1.16	0.76	1.77	0.48	19.22	0.73	141
	Lepercq, 2002	1.76	0.72	4.28	0.21	4.71	0.18	30
	Small, 1987	3.83	1.20	12.24	0.02	2.80	0.11	20
	Abell, 2016	1.08	0.93	1.25	0.30	84.29	3.22	7
	Abell, 2016	0.77	0.61	0.98	0.03	47.89	1.83	7
	Mackin, 2019	0.92	0.85	1.00	0.04	127.57	4.87	61
	Abell, 2016	1.22	1.01	1.48	0.04	63.91	2.44	4
	Abell, 2016	1.03	0.95	1.11	0.45	127.57	4.87	41
	Klemetti, 2012	1.08	1.03	1.13	0.00	146.74	5.60	159
	Abell, 2016	1.01	0.93	1.09	0.80	127.57	4.87	40
	Subgroup Overall	1.03	1.01	1.06	0.03			
Overall		1.06	1.02	1.11	0.00			

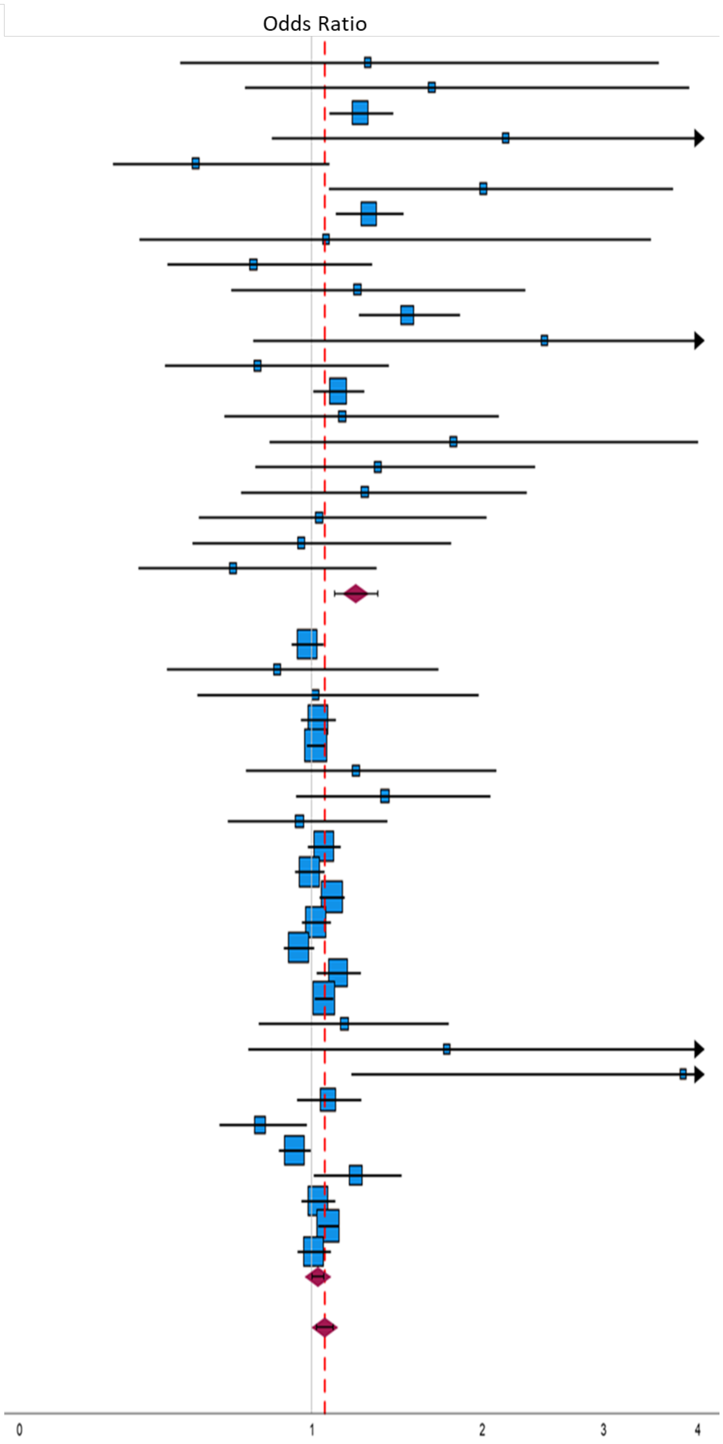

Model: Random-effects model
Heterogeneity: Tau-squared = 0.01, H-squared = 2.90, I-squared = 0.66
Homogeneity: Q = 101.66, df = 45, p-value = 0.00
Test of overall effect size: z = 3.19, p-value = 0.00

Supplementary Figure 1. Forest plot of subgroup analysis – BMI (categorical and continuous variables). PPG: Pregnancy Program Project cohort; CSL: Consortium on Safe Labor cohort

GWG Variable	Study ID	OR	Lower	Upper	p-value	Weight	Weight (%)	N_case
Continuous	Oppermann, 2020	1.06	1.03	1.09	0.00	19.51	6.92	30
	Gutaj, 2017	1.14	1.02	1.28	0.02	18.37	6.52	16
	Castiglioni, 2013	1.13	1.04	1.23	0.00	18.90	6.71	27
	Soholm, 2021	0.98	0.48	1.97	0.95	5.58	1.98	38
	Berk, 1989	1.24	0.66	2.34	0.50	6.46	2.29	55
	Aschwald, 2009	3.21	1.33	7.75	0.01	3.96	1.40	40
	Evers, 2002	1.29	0.85	1.96	0.23	10.32	3.66	141
	Lepercq, 2002	2.46	1.00	6.04	0.05	3.83	1.36	30
	Subgroup Overall	1.11	1.04	1.18	0.00			
Categoric eGWG	Scifres, 2014	1.45	0.57	3.68	0.44	3.59	1.27	25
	Kawakita, 2016	1.27	0.53	3.05	0.59	3.99	1.41	36
	Meek, 2023	4.22	1.13	15.86	0.03	1.97	0.70	14
	Kawakita, 2016	0.99	0.50	1.96	0.98	5.81	2.06	83
	Meek, 2023	1.09	0.54	2.18	0.81	5.67	2.01	49
	Weschenfelder, 2021	2.33	1.08	5.03	0.03	4.87	1.73	51
	Stogianni, 2019	1.76	0.54	5.73	0.35	2.41	0.86	36
	Scifres, 2014	1.84	0.97	3.49	0.06	6.33	2.25	111
	Lepercq, 2010	2.32	1.11	4.84	0.03	5.20	1.84	148
	Secher, 2014	1.13	0.53	2.41	0.75	4.98	1.77	63
	Kawakita, 2016	1.25	0.63	2.48	0.52	5.78	2.05	171
	Meek, 2023	1.73	0.86	3.48	0.13	5.58	1.98	92
	Stogianni, 2019	2.32	0.70	7.69	0.17	2.35	0.83	22
	McWhorter-PPG, 2018	1.57	0.93	2.66	0.09	8.07	2.86	134
	McWhorter-CSL, 2018	1.62	0.97	2.71	0.07	8.32	2.95	131
	Morrens, 2016	1.95	1.08	3.53	0.03	7.03	2.49	117
	Scifres, 2014	3.05	1.74	5.36	0.00	7.49	2.66	53
	Secher, 2014	2.23	1.02	4.85	0.04	4.79	1.70	62
	Hauffe, 2019	1.89	1.02	3.51	0.04	6.65	2.36	136
	Ladfors, 2017	3.53	1.25	9.94	0.02	3.02	1.07	111
	Alexander, 2019	1.19	0.99	1.43	0.06	16.70	5.92	59
	Meek, 2023	1.76	0.89	3.48	0.11	5.78	2.05	55
	Scifres, 2014	2.25	1.29	3.90	0.00	7.69	2.73	36
	Kawakita, 2016	2.78	1.23	6.29	0.01	4.45	1.58	48
	Scifres, 2014	0.62	0.18	2.13	0.45	2.25	0.80	11
	Secher, 2014	0.85	0.05	13.97	0.91	0.48	0.17	62
	Kawakita, 2016	1.47	0.55	3.92	0.44	3.32	1.18	29
	Meek, 2023	3.23	0.63	16.62	0.16	1.34	0.47	8
	Scifres, 2014	1.15	0.52	2.55	0.73	4.61	1.64	34
	Secher, 2014	0.49	0.11	2.15	0.34	1.61	0.57	62
	Kawakita, 2016	1.18	0.64	2.19	0.60	6.65	2.36	120
	Meek, 2023	1.22	0.53	2.79	0.64	4.35	1.54	28
	Scifres, 2014	0.79	0.42	1.49	0.47	6.46	2.29	71
	Secher, 2014	0.94	0.35	2.52	0.90	3.29	1.17	62
	Meek, 2023	1.10	0.52	2.32	0.81	5.06	1.79	37
	Scifres, 2014	0.49	0.23	1.06	0.07	4.88	1.73	33
	Secher, 2014	1.30	0.21	8.07	0.78	1.09	0.38	62
	Kawakita, 2016	2.31	1.22	4.36	0.01	6.41	2.27	74
	Meek, 2023	0.94	0.42	2.08	0.87	4.61	1.64	31
	Subgroup Overall	1.50	1.31	1.73	0.00			
Overall		1.41	1.26	1.59	0.00			

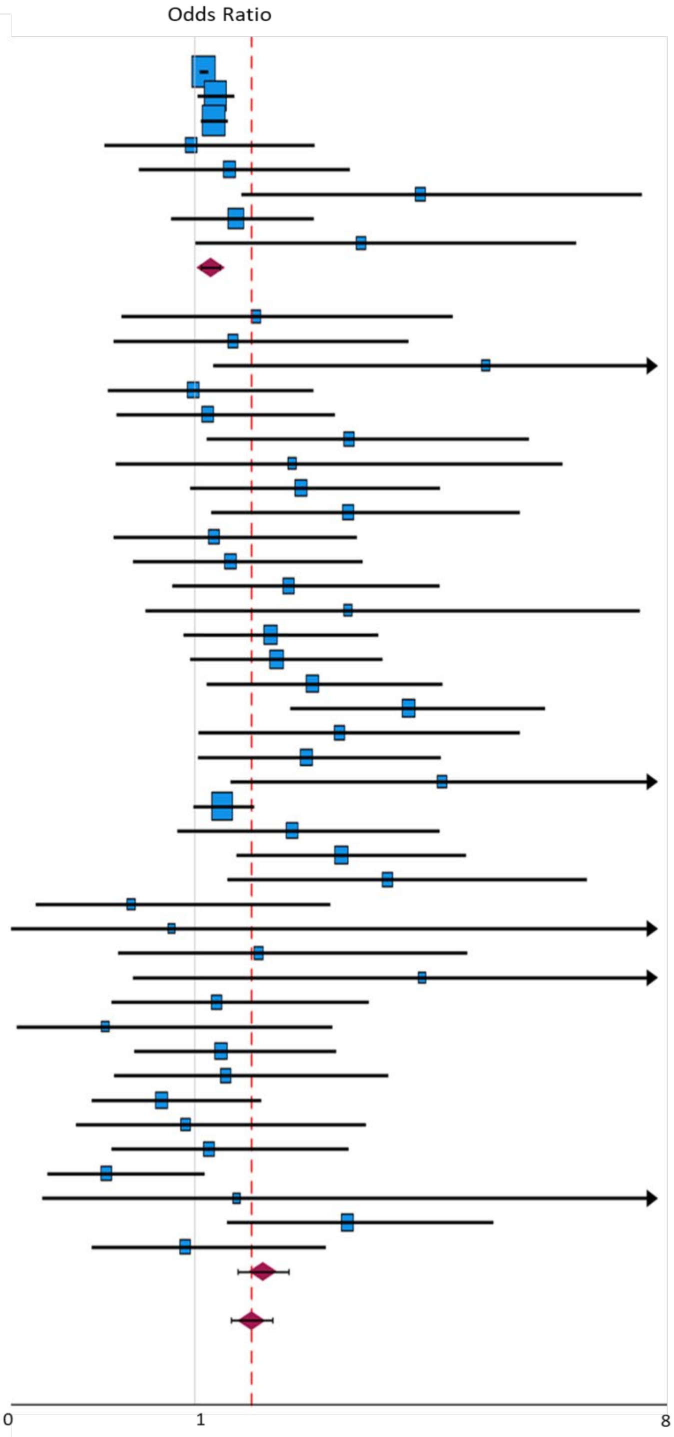

Model: Random-effects model

Heterogeneity: Tau-squared = 0.05, H-squared = 3.44, I-squared = 0.71

Homogeneity: Q = 95.19, df = 46, p-value = 0.00

Test of overall effect size: z = 5.79, p-value = 0.00

Supplementary Figure 2. Forest plot of subgroup analysis – GWG (categorical and continuous variables); eGWG: excessive gestational weight gain; PPG: Pregnancy Program Project cohort; CSL: Consortium on Safe Labour cohort; LGA: Large for gestational age; SGA: Small for gestational age; RDS: Respiratory distress syndrome; NICU: Neonatal intensive care unit

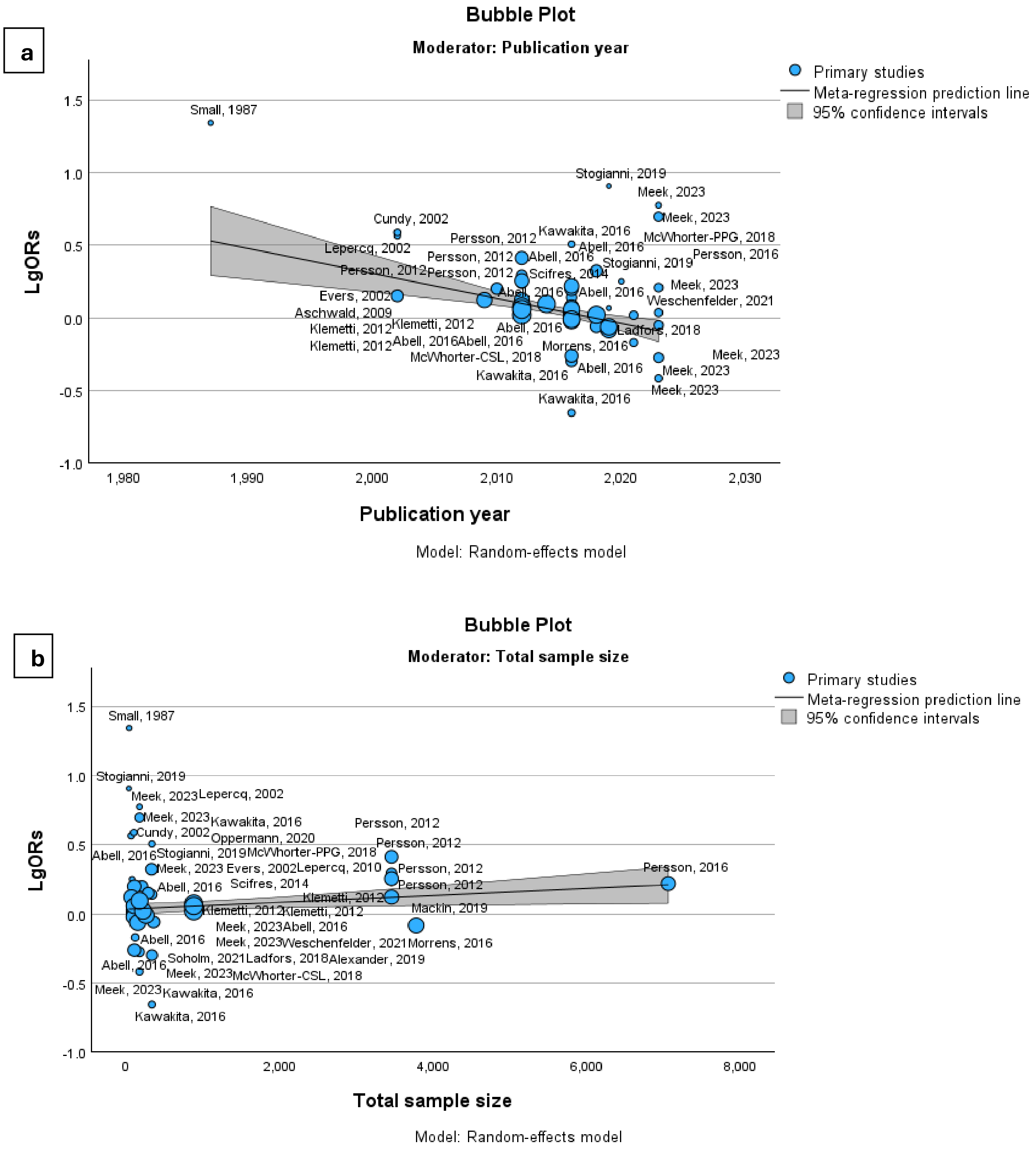

Supplementary Figure 3. Bubble plot of the association between BMI and perinatal outcomes: (a) showing the role of study publication year in the heterogeneity of the study results; (b) showing the role of the sample size in the heterogeneity of the study results

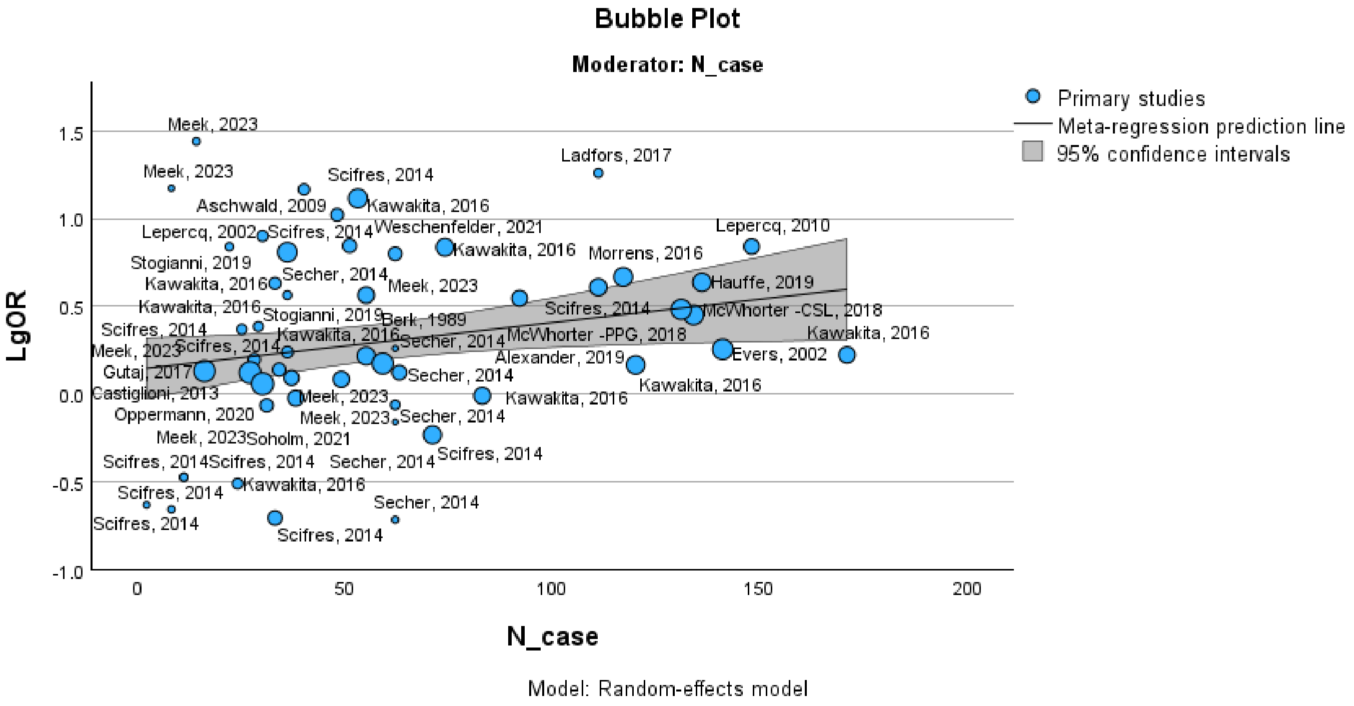

Supplementary Figure 4. Bubble plot of the association between GWG and perinatal outcomes: (a) showing the role of the number of cases in the heterogeneity of the study results

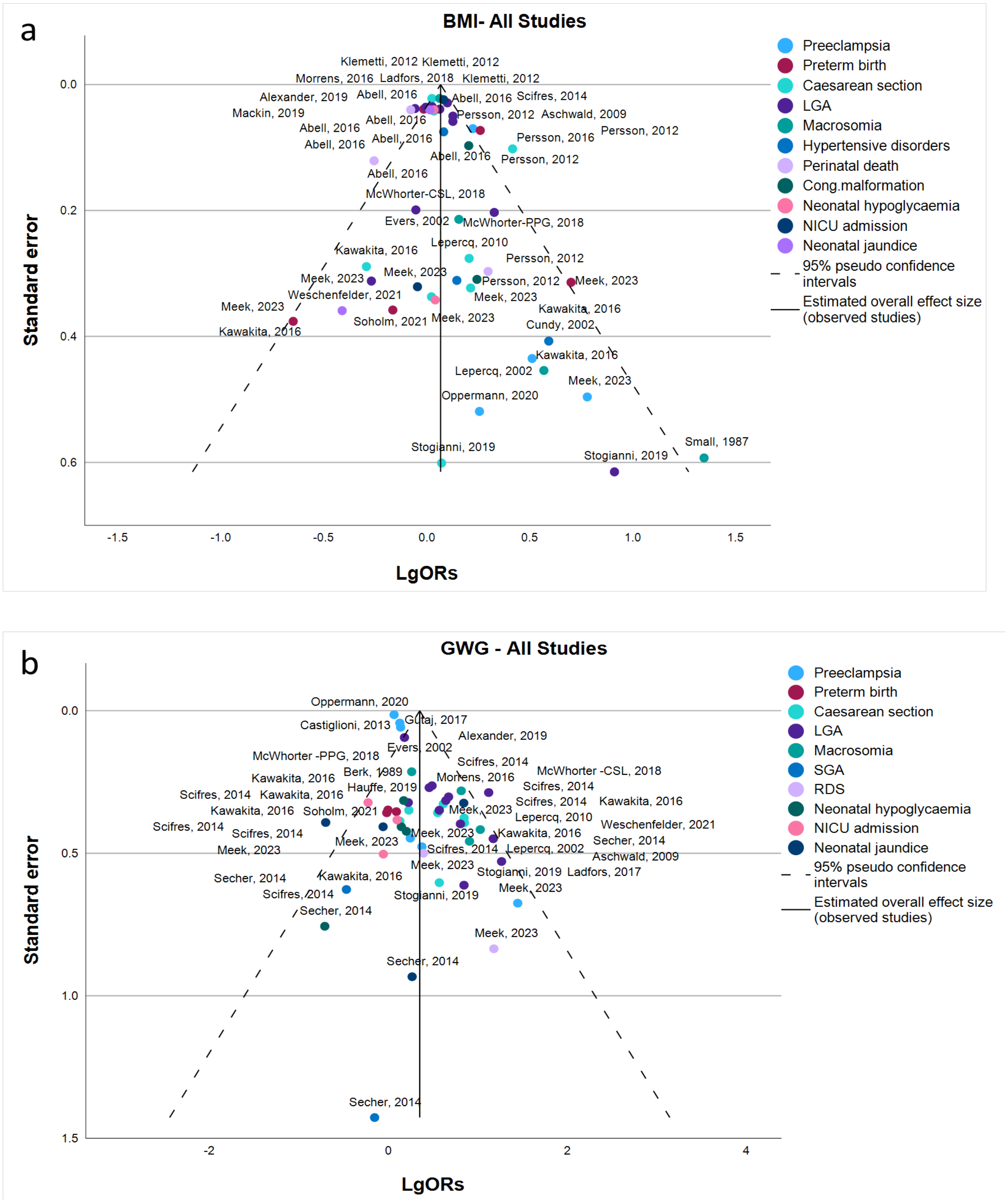

Supplementary Figure 5. Funnel plots of the studies included in the meta-analysis, specifically (a) studies assessing BMI and (b) studies examining GWG in association with perinatal outcomes. PPG: Pregnancy Program Project cohort; CSL: Consortium on Safe Labor cohort; LGA: Large for gestational age; SGA: Small for gestational age; RDS: Respiratory distress syndrome; NICU: Neonatal intensive care unit

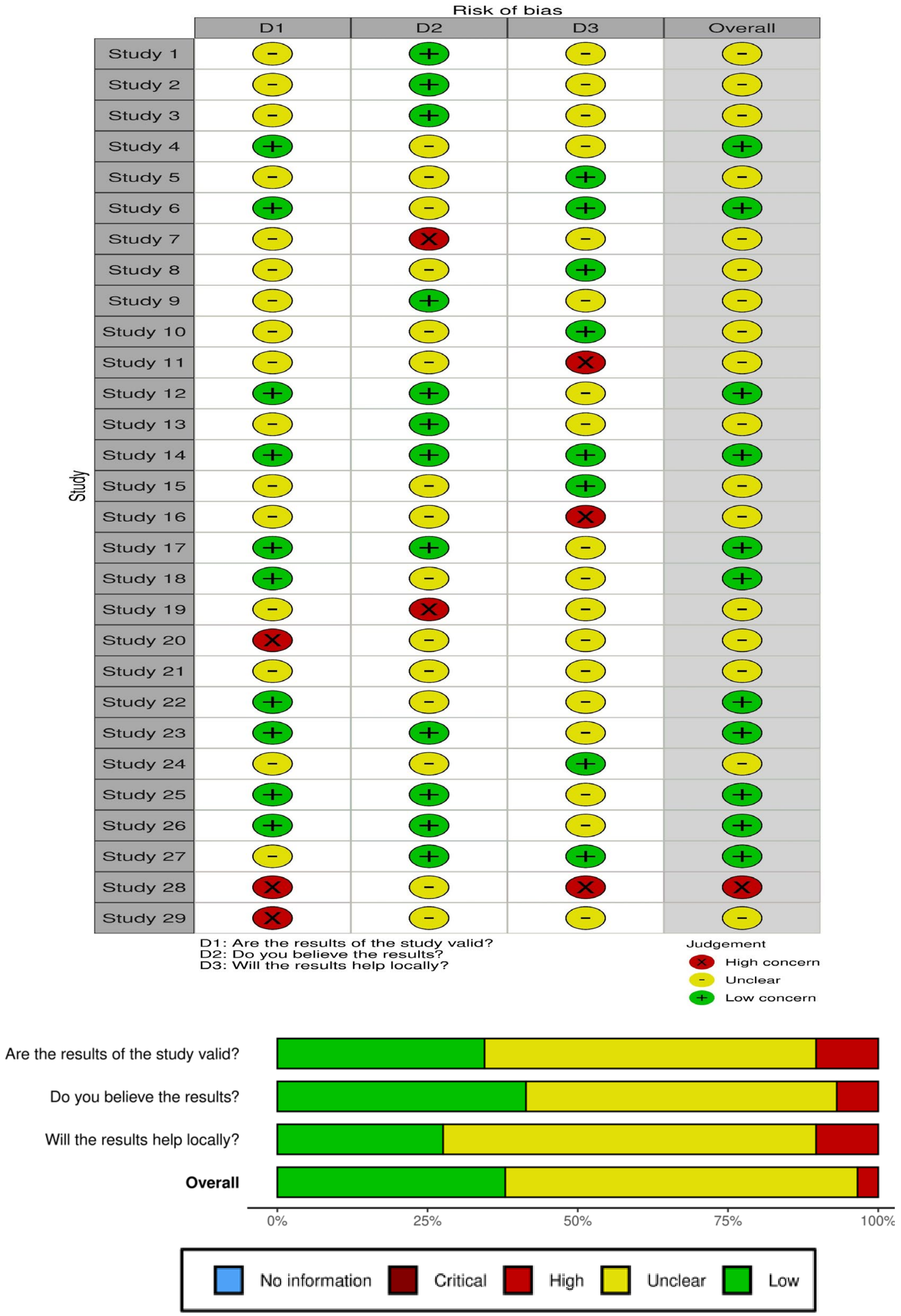

Supplementary Figure 6: Risk of bias appraisal in the primary studies

Supplementary References List (1-29; studies included in this systematic review and meta-analysis)

1. Weschenfelder F, Herrmann E, Lehmann T, Schleußner E, Kloos C, Battfeld W, et al. Predictors of a successful vaginal delivery in women with type 1 diabetes: a retrospective analysis of 20 years. Arch Gynecol Obstet. 2022;305(6):1445–52.
2. Oppermann MLDR, Alessi J, Hirakata VN, Wiegand DM, Reichelt AJ. Preeclampsia in women with pregestational diabetes–a cohort study. Hypertens Pregnancy. 2020;39(1):48–55.
3. Stogianni A, Lendahls L, Landin-Olsson M, Thunander M. Obstetric and perinatal outcomes in pregnancies complicated by diabetes, and control pregnancies, in Kronoberg, Sweden. BMC Pregnancy Childbirth. 2019;19(1):1–10.
4. McWhorter KL, Bowers K, Dolan LM, Deka R, Jackson CL, Khoury JC. Impact of gestational weight gain and prepregnancy body mass index on the prevalence of large-for-gestational age infants in two cohorts of women with type 1 insulin-dependent diabetes: A cross-sectional population study. BMJ Open. 2018;8(3):1–10.
5. Abell SK, Boyle JA, de Courten B, Knight M, Ranasinha S, Regan J, et al. Contemporary type 1 diabetes pregnancy outcomes: Impact of obesity and glycaemic control. Med J Aust. 2016;205(4):162–7.
6. Morrens A, Verhaeghe J, Vanhole C, Devlieger R, Mathieu C, Benhalima K. Risk factors for large-for-gestational age infants in pregnant women with type 1 diabetes. BMC Pregnancy Childbirth. 2016;16(1):1–8.
7. Scifres CM, Feghali MN, Althouse AD, Caritis SN, Catov JM. Effect of excess gestational weight gain on pregnancy outcomes in women with type 1 diabetes. Obstet Gynecol. 2014;123(6):1295–302.
8. Kawakita T, Bowers K, Coviello E, Miodovnik M, Ehrlich S, Rosenn B, et al. Prepregnancy Weight in Women with Type i Diabetes Mellitus: Effect on Pregnancy Outcomes. Am J Perinatol. 2016;33(13):1300–5.
9. Klemetti M, Nuutila M, Tikkanen M, Kari MA, Hiilesmaa V, Teramo K. Trends in maternal BMI, glycaemic control and perinatal outcome among type 1 diabetic pregnant women in 1989-2008. Diabetologia. 2012;55(9):2327–34.
10. Lepercq J, Le Meaux JP, Agman A, Timsit J. Factors Associated With Cesarean Delivery in Nulliparous Women With Type 1 Diabetes. Obstet Gynecol. 2010 May;115(5):1014–20.
11. Secher AL, Parellada CB, Ringholm L, Ásbjörnsdóttir B, Damm P, Mathiesen ER. Higher gestational weight gain is associated with increasing offspring birth weight independent of maternal glycemic control in women with type 1 diabetes. Diabetes Care. 2014;37(10):2677–84.

12. Søholm JC, Vestgaard M, Ásbjörnsdóttir B, Do NC, Pedersen BW, Storgaard L, et al. Potentially modifiable risk factors of preterm delivery in women with type 1 and type 2 diabetes. Diabetologia. 2021;64(9):1939–48.

13. Kawakita T, Bowers K, McWhorter K, Rosen B, Adams M, Miodovnik M, et al. Characterizing Gestational Weight Gain According to Institute of Medicine Guidelines in Women with Type 1 Diabetes Mellitus: Association with Maternal and Perinatal Outcome. Am J Perinatol. 2016;33(13):1266–72.

14. Hauffe F, Schaefer-Graf UM, Fauzan R, Schohe AL, Scholle D, Sedlacek L, et al. Higher rates of large-for-gestational-age newborns mediated by excess maternal weight gain in pregnancies with Type 1 diabetes and use of continuous subcutaneous insulin infusion vs multiple dose insulin injection. Diabet Med. 2019;36(2):158–66.

15. Persson M, Cnattingius S, Wikström AK, Johansson S. Maternal overweight and obesity and risk of pre-eclampsia in women with type 1 diabetes or type 2 diabetes. Diabetologia. 2016;59(10):2099–105.

16. Gutaj P, Zawiejska A, Mantaj U, Wender-Ożegowska E. Determinants of preeclampsia in women with type 1 diabetes. Acta Diabetol. 2017;54(12):1115–21.

17. Ladfors L, Shaat N, Wiberg N, Katasarou A, Berntorp K, Kristensen K. Fetal overgrowth in women with type 1 and type 2 diabetes mellitus. PLoS One. 2017;12(11):1–11.

18. Alexander LD, Tomlinson G, Feig DS. Predictors of Large-for-Gestational-Age Birthweight Among Pregnant Women With Type 1 and Type 2 Diabetes: A Retrospective Cohort Study. Can J Diabetes. 2019;43(8):560–6.

19. Evers I, De Valk H, Mol B, Ter Braak E, Visser G. Macrosomia despite good glycaemic control in Type I diabetic pregnancy; results of a nationwide study in The Netherlands. Diabetologia. 2002;45(11):1484–9.

20. Castiglioni MT, Valsecchi L, Cavoretto P, Pirola S, Di Piazza L, Maggio L, et al. The risk of preeclampsia beyond the first pregnancy among women with type 1 diabetes parity and preeclampsia in type 1 diabetes. Pregnancy Hypertens. 2014;4(1):34–40.

21. Berk MA, Mimouni F, Miodovnik M, Hertzberg V, Valuck J. Macrosomia in Infants of Insulin-Dependent Diabetic Mothers. Obstet Gynecol Surv. 1990 Apr;45(4):247.

22. Lepercq J, Hauguel-De Mouzon S, Timsit J, Catalano PM. Fetal macrosomia and maternal weight gain during pregnancy. Diabetes Metab. 2002 Sep;28(4 Pt 1):323–8.

23. Meek CL, Stewart ZA, Feig DS, Furse S, Neoh SL, Koulman A, et al. Metabolomic insights into maternal and neonatal complications in pregnancies affected by type 1 diabetes. Diabetologia. 2023 Nov 1;66(11):2101–16.

24. Persson M, Pasupathy D, Hanson U, Westgren M, Norman M. Pre-pregnancy body mass index and the risk of adverse outcome in type 1 diabetic pregnancies:

a population-based cohort study. BMJ Open. 2012 Feb 14;2(1):e000601.

25. Mackin ST, Nelson SM, Wild SH, Colhoun HM, Wood R, Lindsay RS. Factors associated with stillbirth in women with diabetes. Diabetologia. 2019 Oct 1;62(10):1938.

26. Cnattingius S, Lindam A, Persson M. Risks of asphyxia-related neonatal complications in offspring of mothers with type 1 or type 2 diabetes: the impact of maternal overweight and obesity. Diabetologia. 2017 Jul 1;60(7):1244–51.

27. Aschwald CL, Catanzaro RB, Weiss EP, Gavard JA, Steitz KA, Mostello DJ. Large-for-gestational-age infants of type 1 diabetic mothers: an effect of preprandial hyperglycemia? Gynecol Endocrinol. 2009 Oct 15;25(10):653–60.

28. Cundy T, Slee F, Gamble G, Neale L. Hypertensive disorders of pregnancy in women with Type 1 and Type 2 diabetes. Diabet Med. 2002;19:482–9.

29. Small M, Cameron A, Lunan CB, MacCuish AC. Macrosomia in pregnancy complicated by insulin-dependent diabetes mellitus. Diabetes Care. 1987;10(5):594–9.
